# The ERP correlates of self-knowledge in ageing

**DOI:** 10.3758/s13421-021-01225-7

**Published:** 2021-08-25

**Authors:** Annick F. N. Tanguay, Ann-Kathrin Johnen, Ioanna Markostamou, Rachel Lambert, Megan Rudrum, Patrick S. R. Davidson, Louis Renoult

**Affiliations:** 1 School of Psychology, University of Ottawa, Ottawa, ON Canada; 2 School of Psychology, University of East Anglia, Norwich, UK; 3 School of Life and Medical Sciences, University of Hertfordshire, Hatfield, UK

**Keywords:** Episodic memory, Semantic memory, Ageing, Event-related potentials

## Abstract

Self-knowledge is a type of personal semantic knowledge that concerns one’s self-image and personal identity. It has most often been operationalized as the summary of one’s personality traits (“I am a stubborn person”). Interestingly, recent studies have revealed that the neural correlates of self-knowledge can be dissociated from those of general semantic and episodic memory in young adults. However, studies of “dedifferentiation” or loss of distinctiveness of neural representations in ageing suggest that the neural correlates of self-knowledge might be less distinct from those of semantic and episodic memory in older adults. We investigated this question in an event-related potential (ERP) study with 28 young and 26 older adults while they categorised personality traits for their self-relevance (self-knowledge conditions), and their relevance to certain groups of people (general semantic condition). Participants then performed a recognition test for previously seen traits (episodic condition). The amplitude of the late positive component (LPC), associated with episodic recollection processes, differentiated the self-knowledge, general semantic, and episodic conditions in young adults, but not in older adults. However, in older adults, participants with higher composite episodic memory scores had more differentiated LPC amplitudes across experimental conditions. Moreover, consistent with the fact that age-related neural dedifferentiation may be material and region specific, in both age groups some differences between memory types were observed for the N400 component, associated with semantic processing. Taken together, these findings suggest that declarative memory subtypes are less distinct in ageing, but that the amount of differentiation varies with episodic memory function.

The distinction between episodic and semantic memory (Tulving, 1972; see also Herrmann, 1982) remains of central importance in Cognitive Neuroscience today. Episodic memory pertains to personal and contextually unique events (I remember reading *1984* at Hyde Park yesterday), whereas semantic memory contains culturally shared, acontextual factual information (*1984* is the title of a book written by George Orwell). The distinction between these two types of declarative memory is supported by a vast amount of behavioural, functional neuroimaging, and neuropsychological research (Addis, 2018; Irish, 2019; Renoult et al., 2019; Renoult & Rugg, 2020; Tulving, 2002) and these two types of memory have been studied extensively in young and older adults. However, recent data also suggest that the relations between semantic and episodic memory may be more complex than previously thought, opening the door to new models of categorization and memory. Not only do semantic and episodic memory seem to interact more than commonly thought (Greenberg & Verfaellie, 2010; Renoult et al., 2015), but also other forms of declarative memory collectively referred to as semantic autobiographical memory or *personal semantics* have recently gained increased attention (Acevedo-Molina et al., 2020; Grilli et al., 2018; Grilli & Verfaellie, 2014, 2015; Irish, 2019; Martinelli, Sperduti, & Piolino, 2013b; Renoult et al., 2012; Renoult et al., 2015; Renoult et al., 2016; Tanguay et al., 2018; Tanguay et al., 2020). Personal semantics refers to the knowledge of one’s past, and knowledge of facts about oneself. The paradox of personal semantics is that it is highly personal (like episodic memory), yet detached from its context of acquisition (like semantic memory). Personal semantics has been operationalized in different ways in the literature. This includes knowledge of autobiographical facts (“I was born on the 25th of June like Orwell”), self-knowledge about one’s traits and identity (“I am a fast reader”), or knowledge of repeated or extended events (“I used to study Orwell’s work at secondary school”; reviewed in Renoult et al., 2012; see also Renoult et al., 2020). Early descriptions assumed that personal semantics was part of semantic memory. However, more recent evaluations of this literature (Grilli & Verfaellie, 2014; Renoult et al., 2012) suggest that this view was too simplistic: whereas some forms of personal semantics—such as autobiographical facts—appear to have neural correlates similar to semantic memory, others—such as memories of repeated events—have neural correlates that are similar to those of episodic memory. This more recent view highlighted that personal semantics had not been well integrated within the framework of declarative memory, but also that there was a critical need for studies comparing personal semantics alongside *both* semantic and episodic memory. Recent lesion and electrophysiological studies indeed suggest that the neural correlates of some forms of personal semantics can be dissociated from (general) semantic memory (Grilli et al., 2018; Grilli & Verfaellie, 2014, 2016; Klein & Lax, 2010; Marquine et al., 2016; Renoult et al., 2015; Renoult et al., 2016; Tanguay et al., 2018; Tanguay et al., 2020). Self-knowledge is a particularly interesting type of personal semantics in this context, as some studies have revealed that it can be dissociated from both semantic and episodic memory (Klein & Lax, 2010; Marquine et al., 2016; Tanguay et al., 2018; Tanguay et al., 2020; Tulving, 1993). Self-knowledge is related to self-image and personal identity and has been most often operationalized as the summary of one’s personality traits (“I am a stubborn person”). However, even though self-knowledge is generally considered to be the most abstract type of personal semantics and to have a greater similarity to semantic than episodic memory (Renoult et al., 2012), its relation to semantic and episodic memory is still unclear because studies have most often compared self-knowledge only to another type of memory (e.g., semantic memory) or examined it in patients with a deficit in a single domain (i.e., semantic or episodic memory). Very few studies have examined the neural correlates that underlie distinctions between self-knowledge, semantic, and episodic memory.

Another relatively unexplored issue is whether personal semantics mainly concerns knowledge of facts and events from our past and present, or also applies to the future (but see Conway et al., 2019, for a recent overview). A number of studies have reported that people also possess knowledge about personal facts and events that they anticipate happening in the future (e.g., D’Argembeau & Demblon, 2012; D’Argembeau & Mathy, 2011). However, it is not perfectly clear whether thinking about these possible selves (Markus & Nurius, 1986) or “temporally extended selves” (Prebble et al., 2013) relies on similar neural substates as thinking about our present self. In the case of self-knowledge, there is evidence that medial prefrontal regions are more active when considering present as compared with past or future selves (D’Argembeau et al., 2008; D’Argembeau et al., 2010), but that other regions like the inferior parietal cortex are more active when thinking about temporally distant selves (D’Argembeau et al., 2010; see also Nyberg et al., 2010). A recent event-related potential (ERP) study observed similar N400 amplitudes, reliably associated with semantic processing (Kutas & Federmeier, 2011), whether participants thought about their present traits or about their past or future self (Tanguay et al., 2018). However, the amplitude of the LPC, typically associated with episodic recollection (Wilding & Ranganath, 2012), was larger when participants considered their past or future traits than their present selves, and these amplitudes were undistinguishable from those elicited in an episodic recognition task (Tanguay et al., 2018; see also Tanguay et al., 2020).

These results suggest that the neural correlates of self-knowledge differ in relation to temporal perspective in young adults. All time perspectives appear to involve semantic memory to some degree but thinking about the self in time would also involve episodic memory, as suggested by the modulations of the LPC (Tanguay et al., 2018; Tanguay et al., 2020). Tulving (2002, 2005) has described the crucial role of episodic memory to mentally travel in one’s own past or future. In contrast, semantic knowledge is thought to be “actualized” in the present moment (Tulving, 1983), but would not be completely atemporal in the sense that it includes knowledge about time and about “possible future worlds” (Tulving, 2005). This is consistent with studies of amnesic patients who are able to list relevant issues about the future, presumably due to their preserved semantic knowledge, but provide impoverished descriptions when asked to elaborate on these descriptions (Race et al., 2013). The importance of this factual knowledge about the future is also demonstrated in semantic dementia patients, suffering from a severe impairment in semantic memory. Indeed, these patients were shown to have difficulties constructing detailed future scenarios, despite relatively preserved episodic memory (Irish et al., 2011; Irish & Piguet, 2013). This has been interpreted as suggesting that semantic memory would provide the “scaffolding” necessary to construct and simulate future events (Irish et al., 2011), consistent with Tulving’s idea that episodic memory operations typically depend on semantic memory (Tulving, 2002). Semantic and episodic memory thus both typically contribute to thinking about our future selves. This entails that, depending on the task or the situation, one might rely to different degrees on general and personal knowledge, and on episodic memory, to reflect on their personal identity.

Studies of age differences in declarative memory have repeatedly reported a decline of episodic memory in ageing (Alghamdi & Rugg, 2020; Cansino, 2009; Tromp et al., 2015) but generally preserved semantic memory function, as assessed for instance by comprehension or general knowledge tests (Irish et al., 2011; Piolino et al., 2003) or via the extraction of semantic details within autobiographical narratives (Levine et al., 2002; St Jacques & Levine, 2007). Very little is known about personal semantics and how its neural correlates differ from those of general semantic and episodic memory in healthy ageing, although this may offer significant new avenues for clinical diagnosis and memory rehabilitation. For instance, as it has been demonstrated that individuals with impaired episodic memory could be trained to rely on personal semantics to compensate for their deficits (Pauly-Takacs et al., 2011; see also Grilli & Ryan, 2020), evaluating preserved subtypes of personals semantics might be a useful approach in memory-impaired individuals. Behavioural research suggests that personal semantics is more resilient to the ageing process than episodic memory (Abram et al., 2014; Martinelli, Sperduti, & Piolino, 2013b; Melendez et al., 2018; Piolino et al., 2003), sometimes somewhat affected (Piolino et al., 2003; Wank et al., 2020), and sometimes unimpaired (Abram et al., 2014; Martinelli, Anssens, et al., 2013a; Melendez et al., 2018) or even enhanced (Acevedo-Molina et al., 2020; Renoult et al., 2020) in older adults compared with younger adults. Self-concept clarity, such as confidence in trait judgements, increases from young adulthood to middle adulthood, but decreases in older age (Lodi-Smith et al., 2017). Limitations in social roles and activities (e.g., due to illness) appear to explain the lower self-concept clarity (Lodi-Smith et al., 2017). There might be age differences in personal semantics when these depend on the medial temporal lobe, such as when they are attached to a spatiotemporal context (Grilli & Verfaellie, 2014, 2016). Characteristics of personal semantics relate with positive outcomes in older adults: For example, higher personal semantics scores are related to identity strength (Haslam et al., 2011). Generating more positive personal semantics is also related with a more positive self-concept (Martinelli et al., 2013a, b), and with well-being (Rathbone et al., 2015).

A number of studies have described a “*dedifferentiation”* or loss of distinctiveness of neural representations in ageing (Cabeza et al., 2018; Koen et al., 2020; Koen & Rugg, 2019; Li et al., 2001). This dedifferentiation has sometimes been interpreted as reflecting an increase in “neural noise”, potentially due to disruption of neuromodulatory systems, or to an imbalance between excitation and inhibition processes (Fornito et al., 2015). Age-related dedifferentiation reflects decreased selectivity of neural activity, and is typically operationalised as a reduced difference in activity in older adults between preferred and nonpreferred stimulus categories for a given brain region (Koen & Rugg, 2019). However, the fact that age-related neural dedifferentiation appears to be material and region specific seems incompatible with interpretations related to a general change in signal to noise in ageing. For instance, while consistent evidence of category-level dedifferentiation has been reported for faces (in the fusiform face area) and for scenes (in the parahippocampal place area), dedifferentiation is much less commonly observed in studies presenting images of objects (reviewed in Koen et al., 2020). Interestingly, recent fMRI studies have reported less distinct item-specific representation in older as compared with younger adults during episodic encoding (Zheng et al., 2018) and during episodic recall (St-Laurent et al., 2014), but also less distinct activations when comparing the neural correlates of semantic and episodic memory (Park et al., 2004; St-Laurent et al., 2011). Similar observations of a dedifferentiation or loss of distinctiveness of neural representations in ageing has been reported in ERP studies (Boutet et al., 2020; Clawson et al., 2017; Galdo-Alvarez et al., 2009; Mott et al., 2014). For example, Boutet et al. (2020), observed less selective and less lateralised N170 amplitudes to faces in older than in young adults. Mott et al. (2014) reported less distinct P300 amplitudes in ageing using a task that examines attentional processes, the oddball task. More precisely, the P300 amplitudes for target (i.e., infrequent stimuli that required a response) and standards (i.e., frequent stimuli that did not require a response) were less distinct in ageing, and the amount of differentiation was negatively associated to age (being reduced in middle age, as compared with young adults, and further reduced in older adults; Mott et al., 2014). However, ERP studies on dedifferentiation in ageing have rarely considered the domain of declarative memory (but see Wolk et al., 2009). A dedifferentiation of memory types in ageing could also apply to personal semantics, its neural correlates being potentially less distinguishable from those of semantic and episodic memory as compared with what is observed in younger adults (Renoult et al., 2016;Tanguay et al., 2018 ; Tanguay et al., 2020). Based on the results that we have reviewed above, a particularly interesting question will be whether differences in the neural correlates of self-knowledge in relation to time perspective (Tanguay et al., 2018; Tanguay et al., 2020) will also be observed in older adults.

Two ERP components are of particular interest in the study of declarative memory: the N400 and the late positive component (LPC), which have been reliably associated with semantic processing (Kutas & Federmeier, 2011) and episodic recollection (Wilding & Ranganath, 2012), respectively. Ageing tends to be associated with a general reduction in amplitude of these ERP components (Wlotko et al., 2010). However, consistent with the relative preservation of semantic memory in ageing, N400 *effects* (the difference between two experimental conditions) are generally found to be similar in young and older adults (Wlotko et al., 2010). ERPs studies have also supported evidence for the preservation of familiarity-based recognition in ageing (Friedman, 2013), where a stimulus may be recognized without retrieval of relevant contextual details (but note that the functional significance of the ERP index of familiarity, or FN400, is still controversial). Finally, consistent with a selective impairment of episodic recollection in ageing, LPC effects are not consistently observed in older adults in episodic recognition memory tasks (Friedman, 2013).

As discussed above, even though self-knowledge is generally considered to be the most abstract type of personal semantics and to have a greater similarity with semantic than episodic memory (Renoult et al., 2012), the question of whether self-knowledge differs from semantic and episodic memory is still unclear. As semantic memory is generally well-preserved in ageing (e.g., Irish et al., 2011; Levine et al., 2002; Piolino et al., 2003; St Jacques & Levine, 2007), we hypothesize that self-knowledge will be a relatively well-preserved form of personal semantics in healthy ageing. However, as mentioned above, it is unclear whether considering self-knowledge across distinct time perspectives would lead to differentiated neural responses in older adults, as observed in young adults. The results of our recent studies with young participants (Tanguay et al., 2018; Tanguay et al., 2020) indeed indicate that the neural bases of self-knowledge partially overlaps with those of semantic memory, but also with episodic memory, but could be differentiated from both. In these studies, LPC amplitudes were maximal for the episodic condition, intermediate for the self-knowledge conditions and minimal for general semantics. In contrast, N400 amplitudes only differentiated self-knowledge from general semantics (Tanguay et al., 2018). To investigate how the neural correlates of self-knowledge are affected in ageing, we recruited a sample of older adults to compare with the young adults of Tanguay et al. (2018; that were reprocessed for the present study). Based on previous studies, we hypothesize that the neural correlates of self-knowledge will be less distinct from those of semantic and episodic memory in older adults. As mentioned above, dedifferentiation is process specific and episodic memory is typically more impaired in ageing than semantic memory. We thus more specifically hypothesized that this dedifferentiation with age will be more apparent for the LPC than for the N400 in the ERP analyses, and that the LPC amplitude would more clearly differentiate time perspective in young adults than in older adults.

Further, older adults may be less sensitive than young adults to factors that influence the proximity of personal semantics to semantic and episodic memory. In young adults, the contextual specificity of personal semantics can make some types appear more like semantic or more like episodic memory. The temporal orientation of self-knowledge—whether it concerns a past or future self versus a present/atemporal self—may be one aspect of contextual specificity, that is, temporal specificity. Further, thinking about a distant self may engage processes associated with temporal distancing because we must make abstraction of the present to think about distant times, like episodic memory (or episodic future thinking). As mentioned above, in our previous studies, we found that temporal distance influenced the LPC amplitude: Past and future self-knowledge produced a larger LPC amplitude than general semantics (Tanguay et al., 2018) and present self-knowledge (Tanguay et al., 2020), but did not significantly differ from episodic memory (Tanguay et al., 2018). Hence, there are conceptual reasons and some data to suggest that thinking about a distant self engages component processes shared with episodic memory (see also Sokol et al., 2017). Here we consider that trait knowledge may be mostly acontextual or generalized across contexts when present-oriented (Tulving, 1983). Episodic processes might help to anchor the self in a richer representation of the future and might contribute to envision distant selves with greater precision. Even though the functional significance of this relation between episodic processes and thinking about a future self is unclear, they could contribute to forming a more differentiated representation of distant selves from the present self while feeling a sense of connection to that future self. Thus, in this study, we aimed to explore whether episodic memory function in older adults related with behavioural differences between the distant times and the present, and with the difference in LPC amplitude between distant times and the present. We also considered other key cognitive functions that are highly sensitive to ageing, such as executive functions (Spreng et al., 2017). In agreement with an episodic memory decline in ageing and our hypotheses, behavioural research indicates that older adults perceive that their traits change less through time compared with young adults (Rutt & Lockenhoff, 2016). Interestingly, in this study, health, personality and cognitive factors (e.g., working memory, processing speed) did not account for this age-difference (Rutt & Lockenhoff, 2016). However, episodic memory function was not examined. In the present study, we thus explored the neural and cognitive basis for age-difference in trait continuity.

## Methods

### Participants

Thirty-two young participants (15 men), with ages ranging from 18 to 33 years (*M*_age_ = 20.97, *SD* = 3.65; one participant did not report their age), and 43 older participants (22 men), with ages ranging from 65 to 86 (*M*_age_ = 73.44, *SD* = 5.51), participated in this study. Young adults had completed an average of 14.84 (*SD* = 2.23) years of education, and older adults completed an average of 14.00 (*SD* = 2.78) years of education (two missing values).

Undergraduate psychology students at the University of East Anglia were recruited through an online system and awarded partial course credit. All other participants were recruited through a participant panel at the School of Psychology of the University of East Anglia, and received £13–15 for their participation (commensurate with study duration). Exclusion criteria consisted of a history of head injury with loss of consciousness longer than 5 min, other neurological or medical conditions known to compromise brain function, and active substance abuse. All participants had normal or corrected-to-normal vision, were English native speakers, and were right-handed. The older adults’ score on the Montreal Cognitive Assessment (MoCA; Nasreddine et al., 2005) was equal to or greater than 23 (*M* = 27.16; *SD* = 1.86). Some research indicates that a cut-off of 26 may be overly stringent and may need to be as low as 20 (Waldron-Perrine & Axelrod, 2012). We included the only two participants with values below 26. Five participants (two young adults and three older adults) did not meet eligibility criteria (i.e., one was left-handed and four for health reasons). We excluded an additional 15 participants (two young adults and 14 older adults) due to a low number of *yes* responses (i.e., indicating that a certain trait applied to them in the past, present, or future or to a specified profession) included in the average (<15), resulting in a sample of 28 young adults and 26 older adults. The main reason for the high level of exclusion for older adults was an increased tendency to move during testing, creating artefacts that sometimes led to the exclusion of a large number of trials. The task included five experimental blocks and even though participants were offered breaks between blocks, it was still long (2 to 2.5 hours in total). Even if these artefacts were constrained to one experimental block (i.e., one experimental condition), data from the other conditions had to be excluded as well due to the nature of the analysis of variance (ANOVA) analysis. The data of the young adults have been reported in Tanguay et al. (2018); they serve as the comparison group for the older adults of this study.

A sample size of 26 older adults allows us to detect an effect of Memory on the LPC amplitude assuming the effect size is half as large as the one of young adults (η_p_^2^ = .08 vs. η_p_^2^ = .16; Tanguay et al., 2018). In G*Power (Version 3.1.9.7; Faul et al., 2007), we specified that effect size with an alpha level of .05, power of .90, 2 group, 5 measurements, a .5 correlation among repeated measures (default), and a nonsphericity correction of 1 (no correction, default), which would require a sample of 20 participants. However, we aimed for a sample size at least similar to the young adults.

### Neuropsychological assessment

In the group of older adults, a comprehensive neuropsychological assessment was carried out within 12 months from the experimental session to assess overall cognitive function, episodic memory, and executive functions. The MoCA (Nasreddine et al., 2005) was used as a screening measure of general cognitive function. We used the Logical Memory type subtest (WMS III UK; Wechsler, 1997) as a measure of episodic memory recall for verbal information. In this task, a short story was read aloud to participants, who then had to recall it immediately and after a 25-min delay. For the assessment of episodic memory recall for visuospatial information, we used the Rey–Osterrieth Complex Figure Test (Osterrieth, 1944; Strauss et al., 2006), in which participants were asked to copy a picture of a complex figure and then to reproduce it from memory type immediately and after a 25-min delay. We averaged the *Z* scores on Logical Memory and Rey–Osterrieth complex figure delayed recall to obtain a composite of episodic memory function (based on Glisky et al., 1995).

Verbal working memory was assessed with the Digit Span (WMS III UK; Wechsler, 1997), in which participants had to repeat a random series of orally presented digits of progressively increasing length in the same order (forward condition) or in reverse order (backward condition). In the Verbal Fluency task (VF; Strauss et al., 2006), we asked participants to generate in 60 seconds as many different words as possible beginning with the letters *F*, *A*, or *S*. We averaged the *Z* scores of the backward Digit Span and the Verbal Fluency to obtain a composite of executive functions (as in Glisky et al., 1995).

Similarly, we derived a composite score for processing speed from the averaged *Z* score on two tasks. Participants connected a series of numbers in ascending order as quickly and accurately as possible in Part A of the Trail Making Test (TMT; Reitan, 1958). For two control measures of the Stroop task (ST; Golden, 1976), participants named the colours of printed words and read the words as quickly and accurately as possible (no conflict).

We omitted the Stroop and Trail Making measure from the executive functions composite score (and rather included Digit Span and Verbal Fluency) for three reasons: First, backward digit span and verbal fluency are typically correlated with one another and load on the same principal component when examined with other executive function and with memory tasks in healthy older adults (Glisky et al., 1995; Glisky et al., 2001). Second, Stroop and Trail Making are not strongly associated with backward digit span and verbal fluency in healthy older adults, perhaps because Stroop and Trail Making Part B place greater demands on inhibitory function (Davidson & Glisky, 2002). Finally, the inclusion of the backward digit span and verbal fluency in an executive functions composite score aligns with prior and ongoing work on memory, cognition, and brain function (e.g., Davidson et al., 2019; Taler et al., 2020).

### Experimental tasks

This study included five memory type conditions: general semantics, episodic memory, and past, present, and future self-knowledge. The experimental paradigm is described in more details in Tanguay et al. (2018). Briefly, in the self-knowledge conditions, participants were asked to indicate if words reflected their past (5 years ago), present, or future (in 5 years) character traits. In the general semantic task, participants were asked to indicate whether the character traits reflected those of most people holding a specific occupation. We attributed twenty traits (half positive, half negative) to each of the four occupations, (i.e., soldiers, priests, lawyers, scientists); the order of these occupation was randomized. For instance, the task would entail judging whether “fearless” reflects the traits of soldiers, whereas being “social” might not be perceived as reflective of scientists. We selected familiar occupations that were strongly associated with some traits (e.g., scientists are inventive), as confirmed with pilot data (Tanguay et al., 2018). After these four conditions, participants completed an episodic recognition memory task, indicating if the word had been presented previously (i.e., target word) or if it was a new word. Participants also provided a confidence rating after each trial.

### Stimuli

Four-hundred words describing people retrieved from Dumas et al. (2002) were classified as either negative or positive (i.e., valence ratings below or above 5 respectively obtained from Warriner, 2013) and included in this study. We generated six word lists, each consisting of 80 words each (40 positive, 40 negative). We randomly assigned three lists to the past, present, and future versions of the self-knowledge task. There was a single list of traits for the general semantic condition to purposefully vary the traits’ relevance to an occupation (e.g., soldier: courageous). Lastly, there were two lists for the episodic memory condition, for old and new words, respectively. We selected words randomly from the general semantic and the three self-knowledge conditions (10 positive, 10 negative traits from each) to form the list of target words in the episodic recognition memory task (80 old, 80 new). All lists were matched on likableness, word frequency (Kucera & Francis, 1967), and word length.

### Procedure

Participants sat approximately 1 m in front of a computer screen and were interviewed about their life circumstances 5 years ago, in the present, and 5 years in the future while the cap was prepared. The experimental tasks were presented with E-Prime 2.0 (Psychology Software Tools, Pittsburgh, PA). Experimental conditions (general semantics, episodic memory, and past, present, and future self-knowledge) were presented in separate blocks with short breaks between blocks to allow participants to rest if necessary. The general semantic condition randomly preceded or followed the three self-knowledge conditions which were also presented in random order. The episodic recognition memory task always ended the study. Trials within each block were presented in random order.

As a general rule, each trial started with a fixation cross of a variable duration (1,500–2,000 ms), after which a trait was shown for 2,000 ms (see Fig. 2). The maximum response time was 3,000 ms, during which people could press 1 or 2 to respond. A “1” signified “I think the word reflects my (past/present/future) traits” and “2” meant “I think the word does not reflect my (past/present/future) traits.” Similarly, in the semantic memory type task, a “1” represented agreement with the statement “I think the word reflects the traits of most people holding the occupation”, and a “2” showed a disagreement. A white screen followed the trait screen for 200 ms, and this sequence of events ended with a blink screen for 1,000 ms (inviting participants to blink, if needed).

Additionally, in the general semantic condition, another self-paced screen was shown every 20 trials indicating a new occupation to make trait judgements about. In the episodic memory condition, participants were asked to respond after the trait was presented to indicate if they had seen the word before (press 1) or not (press 2). They were then also asked to report their confidence (1 = *Quite sure*; 2 = *Relatively sure*; 3 = *Not sure*; based on Renoult et al., 2015). The blink screen was omitted in the episodic memory condition as participants were able to blink while responding, if needed.

### EEG acquisition and preprocessing

The electroencephalogram (EEG) was recorded with a 63-channel active electrode system (Brain Products GmbH) embedded in a nylon cap (10/10 system extended). An additional electrode was placed under the left eye in order to monitor vertical eye movements (lower EOG). The continuous EEG signal was acquired at a 500 Hz sampling rate using an FCz reference. The high filter was set at 250 Hz and the time constant was 10 s. The impedance was kept below 20 kΩ. A vertical EOG was reconstructed offline as the difference between the lower EOG and FP1 activity. A horizontal EOG was constructed by subtracting FT9 from FT10 activity.

Offline analyses were carried out using Brain Vision Analyser 2. Steps and processing parameters were similar to Tanguay et al. (2018), but optimized to retain a maximum number of trials with older adults. Notably, the filtering parameters were .1 to 30Hz (order 2) with a 50 Hz Notch filter (instead of .01 to 30Hz without a notch filter). After filtering, we removed excessively bad channels and performed a semi-automatic data inspection to exclude noisy segments. We excluded frontal channels (AF3, AF3, AF7, AF8, Fp1, Fp2) from the automatic detection of noise as these electrodes are sensitive to blink artifacts. Analyzer highlighted segments with an absolute difference of two contiguous sampling points larger than 75 μV, and a difference between the minimal and maximal voltage group larger than 150 μV within a 200-ms interval. We inspected all data to confirm that all noise was effectively excluded. Component reflecting eye movements were removed using automatic ICA ocular correction (Jung et al., 2000), and the previously removed channels were interpolated using spherical interpolation. An average reference was computed offline and used for all analyses. The EEG was segmented into epochs of 1 s (from −200 ms prior to, to 800 ms after the onset of the words). The 200 ms precue period was used for baseline correction. Trials were rejected after a 200-ms baseline correction if they did not meet the criteria listed above (see automatic detection of noise), now with all channels, and (1) if the voltage group was above 100 μV or below −100 μV, or (2) if the difference between the minimum and maximum voltage group was less than .5 μV for 100 ms. Participants were excluded if the average of any condition had less than 15 trials.

The amplitudes of the N400 and the LPC were measured as the mean of all data points between 250 to 500 ms and 500 to 800 ms, respectively, as in Tanguay et al. (2018). The N400 is typically studied at sagittal or para-sagittal sites, and the LPC at the posterior parietal sites. We included additional sites for the LPC time window (i.e., frontal, sagittal, para-sagittal) as scalp distribution can differ in older adults compared with young adults (e.g., J. H. Ford & Kensinger, 2019; Horne et al., 2020; Newsome et al., 2012). We averaged Cz, CPz, and Pz for the sagittal subset, and averaged C1/C3/CP3 for the left and C2/C4/CP4 for the right hemisphere of the para-sagittal subset, and averaged P1/P3/PO3 for the left and P2/P4/PO4 for right hemisphere of the posterior parietal subset, and also F1/F3/FC3 for the left and F2/F4/FC4 for the right hemisphere of the frontal subset

### Statistical analysis

We ran repeated-measures ANOVAs on behavioural and electrophysiological data. For the self-knowledge (past, present, and future self-knowledge) and the general semantic condition, we focused on reaction times and percentage of yes responses as our behavioural measures while we focused on reaction times, accuracy, sensitivity, and bias for the episodic memory condition. The averaged ERP activity for each memory condition across the ERP-specific time windows was used as our electrophysiological measure. In addition, we analysed the averaged LPC activity for hits and correct rejections for the episodic memory condition only. For the electrophysiological data, 26 participants in the older adults age group were included for which sufficient data was available in all memory type conditions with an average group number of 29.5 trials in each condition (Min = 16, Max = 62). For the young adults age group 28 participants with sufficient trial numbers, on average 34.2 in each condition (Min = 16, Max = 72), were included. Only “yes” responses were retained for these ERP analyses as these suggest participants were sufficiently confident in the presence of a memory type trace. Further, we operationalized episodic memory as correct recognition of old items regardless of confidence. This differed from Tanguay et al. (2018) because we aimed to retain a maximum of older adults in our analyses. In additional analyses, to fully characterize our results, we verified whether hits elicited a larger LPC amplitude than correct rejections.

The task design is identical to Tanguay et al. (2018) and the sample of young adults is the same. For greater details on the task (e.g., stimuli list) and findings for young adults (e.g., P200), we invite the reader to refer to Tanguay et al. (2018). The preprocessing steps and analytical steps differed somewhat to retain a maximum of participants and simplify interpretation because of the added age factor.

## Results

Twenty-eight young participants (13 men), with ages ranging from 18 to 33 years (*M*_age_ = 21.26, *SD* = 3.83; one participant did not report their age), and 26 older participants (15 men), with ages ranging from 65 to 83 (*M*_age_ = 73.50, *SD* = 5.32), were included in the analysis for this study. Young adults had completed an average of 15.00 (*SD* = 2.34) years of education, and older adults completed an average of 14.12 (*SD* = 2.59) years of education (one missing value).

### Behavioural data: Reaction time

**Fig. 1:**
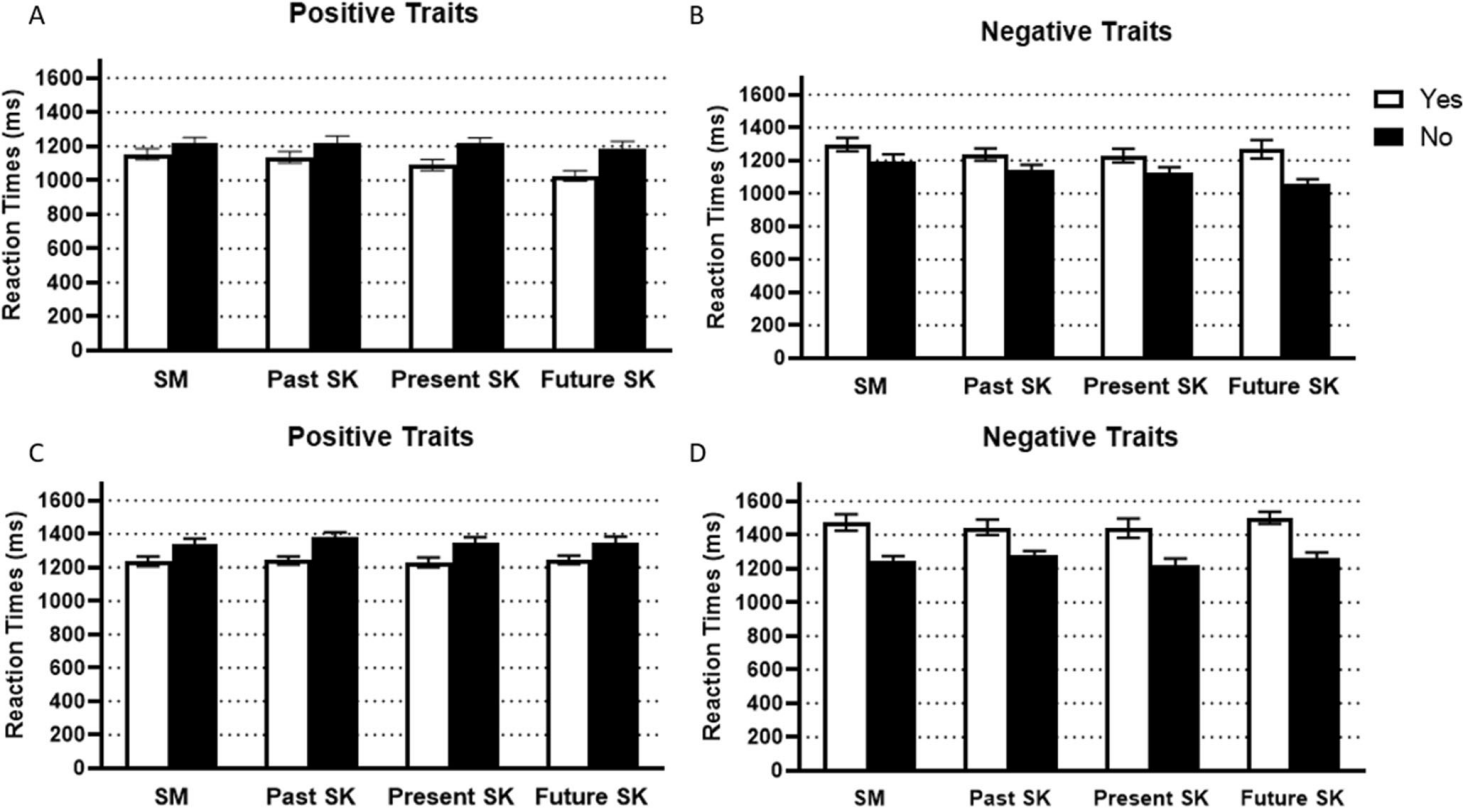
Mean RTs for positive traits (left; **a, c**) and negative traits (right; **b, d**) for young adults (top row; **a, b**) and older adults (bottom row; **c, d**) for yes and no responses in each memory condition. Error bars represent ± 1 *SE*. **a** Mean RTs for positive traits for young adults. **b** Mean RTs for negative traits for young adults. **c** Mean RTs for positive traits for older adults. **d** Mean RTs for negative traits for older adults. SM = semantic memory; SK = self-knowledge

A 2 × 4 × 2 × 2 mixed ANOVA with Age group (between subject factor with two levels: young adults and older adults), Memory type (within subject factor with four levels: general semantic, past self-knowledge, present self-knowledge, and future self-knowledge), Valence (within subject factor with two levels: positive and negative), and Response (within-subject factor with two levels: yes and no) on mean reaction times (see Fig. 1) revealed a significant main effect of Age group, *F*(1, 49) = 14.28, *p* < .001, η_p_^2^ = .23, with faster reaction times for young (*M* = 1175.60, *SE* = 26.73) compared with older adults (*M* = 1326.01, *SE* = 29.50). There were also significant main effects of Valence, *F*(1, 49) = 32.48, *p* < .001, η_p_^2^ = .40, and Response, *F*(1, 49) = 10.08, *p* = .003, η_p_^2^ = .17. For Memory type, the main effect was not significant, *F*(2.44, 119.47) = 1.22, *p* = .302, η_p_^2^ = .02, but there was a significant interaction with Age group, *F*(2.44, 119.47) = 2.87, *p* = .050, η_p_^2^ = .06. Although young adult always responded faster than older adults, the magnitude of the difference depended on the Memory type (general semantics: *p* = .022, η_p_^2^ = .10; past self-knowledge: *p* = .002, η_p_^2^ = .18; present self-knowledge: *p* = .003, η_p_^2^ = .17; future self-knowledge: *p* < .001, η_p_^2^ = .29).

The effect of Valence depended on Memory type, *F*(3, 147) = 2.81, *p* = .042, η_p_^2^ = .05. Participants responded faster to positive traits than negative traits in all conditions (general semantics: *p* < .001, η_p_^2^ = .24; present self-knowledge, *p* = .021, η_p_^2^ = .10; future self-knowledge, *p* < .001, η_p_^2^ = .30), except past self-knowledge where positive and negative traits were not significantly different (*p* = .223, η_p_^2^ = .03). The interactions between Response and Age group, *F*(1, 49) = 5.34, *p* = .025, η_p_^2^ = .10, Response and Valence, *F*(1, 49) = 201.26, *p* < .001, η_p_^2^ = .80, and between Response, Valence, and Age group, *F*(1, 49) = 5.64, *p* = .022, η_p_^2^ = .10, were also significant. We ran repeated-measures ANOVAs separately for each group. The effect of Response depended on Valence for young adults, *F*(1, 27) = 86.36, *p* < .001, η_p_^2^ = .76. Young adults were faster to endorse than to reject a trait if it was positive (*p* < .001, η_p_^2^ = .73), but slower to endorse than to reject a trait if it was negative (*p* < .001, η_p_^2^ = .55). Similarly, the effect of Response depended on Valence for older adults, *F*(1, 22) = 110.71, *p* < .001, η_p_^2^ = .83. Like for young adults, older adults were faster to endorse than reject a trait if it was positive (*p* < .001, η_p_^2^ = .69), but slower to endorse than to reject a trait if it was negative (*p* < .001, η_p_^2^ = .78). The interaction seems to have arisen because the interaction between Response and Valence was stronger in older adults than young adults.

The interactions Memory type, Response, Valence, and Age group, *F*(3, 147) = 2.43, *p* = .068, η_p_^2^ = .05, Memory type, Response, and Age group, *F*(3,147) = 0.16, *p* = .922, η_p_^2^ < .01, Memory and Response, *F*(3, 147) = 1.13, *p* = .338, η_p_^2^ = .02, Memory type, Valence, and Age group, *F*(3, 147) = 0.03, *p* = .992, η_p_^2^ < .01, Valence and Age group, *F*(1, 49) = 1.11, *p* = .298, η_p_^2^ = .02, were not significant.

This mixed ANOVA excluded three older participants because they did not have responses for a condition (e.g., “yes” to having a negative trait in the past), and consequently no reaction time.

### Behavioural data: Percentage of “yes” responses

**Fig. 2:**
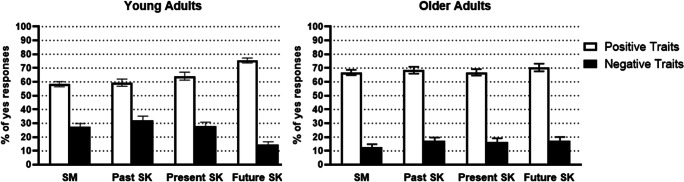
Mean percentage of yes responses for young adults (left) and older adults (right) for positive and negative traits in each memory condition; semantic memory (SM), past self-knowledge (past SK), present self-knowledge (present SK), and future self-knowledge (future SK). Error bars represent ± 1 *SE*

A 2 × 4 × 2 mixed ANOVA with Age group (between subject factor: young adults and older adults), Memory type (within subject factor: general semantic, past self-knowledge, present self-knowledge, and future self-knowledge), and Valence (within subject factor: positive and negative) on percentage of yes responses revealed a main effect of Memory type, *F*(3, 156) = 3.59, *p* = .015, η_p_^2^ = .07, and a main effect of Valence, *F*(1,52) = 496.55, *p* < .001, η_p_^2^ = .91. There was also a significant interaction between Memory type and Valence, *F*(3, 156) = 12.49, *p* < .001, η_p_^2^ = .19, as well as between Memory type, Valence, and Age group, *F*(3, 156) = 11.10, *p* < .001, η_p_^2^ = .18 (see Fig. 2), and data were analysed for each age group separately. For young adults, the percentage of endorsed traits per Memory type depended on Valence, *F*(3, 81) = 20.15, *p* < .001, η_p_^2^ = .43. Briefly, young adults endorsed more positive traits and less negative traits in the future self-knowledge condition compared with all other conditions (i.e., general semantics, past self-knowledge, present self-knowledge, *p*s < .001; full details described in Tanguay et al., 2018). Comparatively, the number of endorsed traits per Valence was not modulated by Memory type for older adults, *F*(3, 75) = 0.30, *p* = .826, η_p_^2^ = .01. Hence, the temporal orientation of self-knowledge or whether knowledge concerned the self or other people did not influence the percentage of endorsed positive and negative traits for older adults. There was, however, a main effect of Valence as can be expected *F*(1, 25) = 253.76, *p* < .001, η_p_^2^ = .91, with more positive traits being endorsed than negative traits. Further, the percentage of endorsed traits did not differ between Memory types for older adults, *F*(3, 75) = 2.49, *p* = .067, η_p_^2^ = .09.

There was also a significant interaction between Valence and Age group, *F*(1, 52) = 10.81, *p* = .002, η_p_^2^ = .17, and data were collapsed across Memory type. Young and older adults endorsed positive traits at similar rates, (*p* = .119, η_p_^2^ = .05), whereas young adults were more likely to endorse negative traits than older adults (*p* < .001, η_p_^2^ = .20). The interaction between Memory type and Age group was not significant, *F*(3, 156) = 0.68, *p* = .567, η_p_^2^ = .01.

### Behavioural data: Recognition memory task

We entered the measures of performance (i.e., reaction times, accuracy, sensitivity, bias) on the recognition memory task in mixed ANOVAs with Age group (between subject: young adults, older adults) and Valence (within subject: positive, negative) as factors. The analyses of reaction times and accuracy also included Response type (within subject factor: hits and correct rejections).

#### Reaction times

**Fig. 3:**
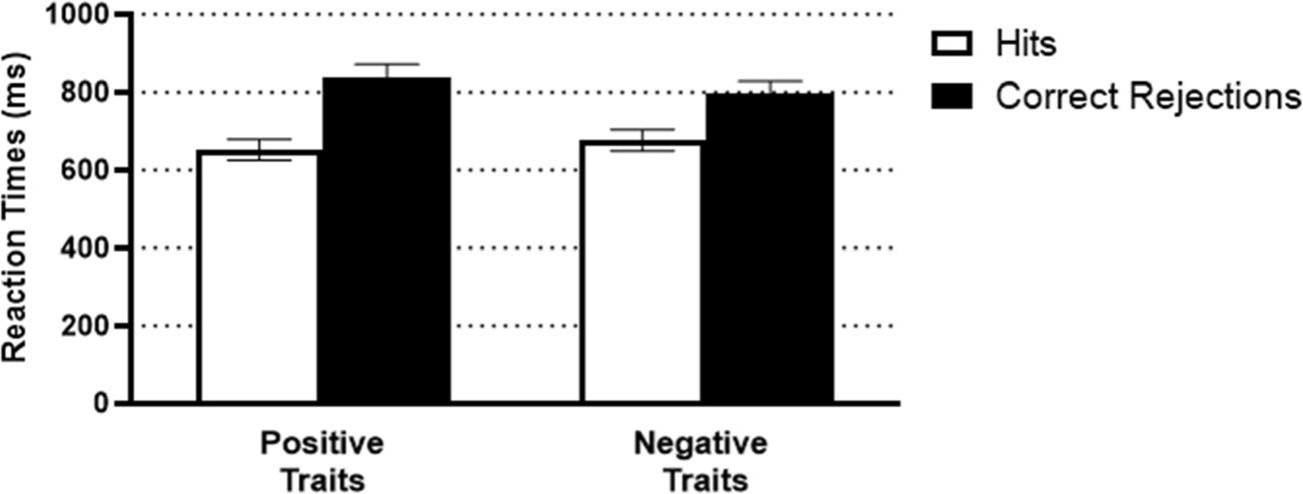
Mean RTs for positive and negative traits for hits and correct rejections. Error bars represent ±1 *SE*

This mixed ANOVA revealed a significant main effect of Age group, *F*(1, 52) = 47.06, *p* < .001, η_p_^2^ = .48 with faster reaction times for young (*M* = 566.39, *SE* = 35.32) compared with older adults (*M* = 915.52, *SE* = 36.65). The main effect of Response, *F*(1, 52) = 41.51, *p* < .001, η_p_^2^ = .44, but not Valence, *F*(1, 52) = 0.39, *p* = .537, η_p_^2^ = .01, was significant. The interaction between Response and Valence was also significant, *F*(1, 52) = 10.68, *p* = .002, η_p_^2^ = .17 (see Fig. 3). Participants had faster response times for negative than positive traits when making correct rejections (*p* = .039, η_p_^2^ = .08), whereas negative and positive traits did not differ for hits (*p* = .129, η_p_^2^ = .04). The other interactions were not significant: Valence and Age group, *F*(1, 52) = 0.002, *p* = .967, η_p_^2^ < .01; Response type and Age group, *F*(1, 52) = 1.20, *p* = .278, η_p_^2^ = .02; Age group, Response type, and Valence, *F*(1, 52) = 0.93, *p* = .341, η_p_^2^ = .02.

#### Accuracy

**Fig. 4:**
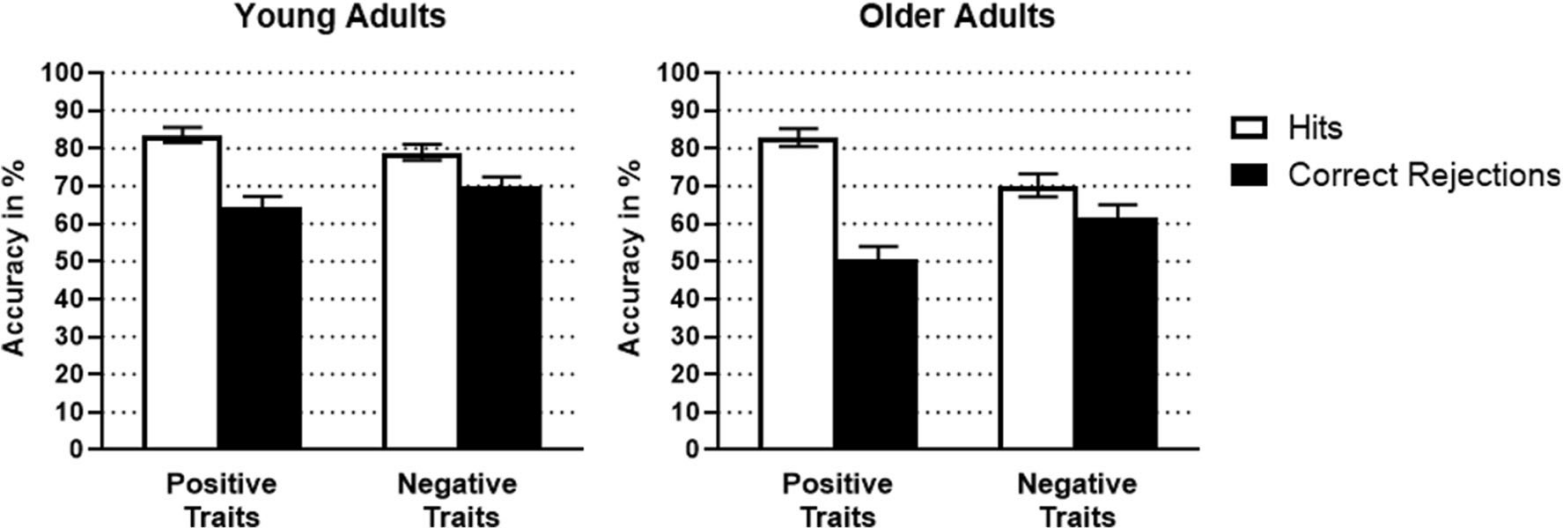
Mean accuracy in percentage for young adults (left) and older adults (right) for positive and negative traits for hits and correct rejections. Error bars represent ±1 *SE*

This mixed ANOVA revealed a significant main effect of Response, *F*(1, 52) = 31.92, *p* < .001, η_p_^2^ = .38, but not Valence, *F*(1, 52) = 0.06, *p* = .806, η_p_^2^ < .01, as well as significant interactions between Response type and Valence, *F*(1, 52) = 101.58, *p* < .001, η_p_^2^ = .66, and between Response type, Valence, and Age group, *F*(1, 52) = 15.34, *p* < .001, η_p_^2^ = .23 (see Fig. 4). We conducted separate ANOVAs for each Age group.

For older adults, the effect of Response type depended on Valence, *F*(1, 25) = 72.36, *p* < .001, η_p_^2^ = .74. Older adults had more hits for positive than negative traits (*p* < .001, η_p_^2^ = .69), and more correct rejections for negative compared with positive traits (*p* < .001, η_p_^2^ = .57). Similarly, Response type and Valence interacted for young adults, *F*(1, 27) = 27.48, *p* < .001, η_p_^2^ = .50. More precisely, young adults had more hits if traits were positive than when they were negative (*p* = .003, η_p_^2^ = .28), and less correct rejection if traits were positive rather than negative (*p* = .004, η_p_^2^ = .27).

The interactions between Valence and Age group, *F*(1, 52) = 0.52, *p* = .473, η_p_^2^ = .01, and between Response type and Age group, *F*(1, 52) = 0.91, *p* = .346, η_p_^2^ = .02, were not significant.

#### Sensitivity (*d'*) and Bias (*c*)

For sensitivity, we found a main effect of Age group, *F*(1, 52) = 14.92, *p* <.001, η_p_^2^ =.22, because sensitivity was higher in young adults (*M* = 1.49, *SE* = .09) compared with older adults (*M* = 1.01, *SE* = 0.09). The main effect of Valence, *F*(1, 52) = 3.29, *p* = .08, η_p_^2^ = .06, and the interaction between Valence and Age group, *F*(1, 52) = 1.04, *p* = .313, η_p_^2^ = .02, were not significant.

The measure of bias produced a main effect of Valence, *F*(1, 52) = 104.78, *p* = .001, η_p_^2^ = .67, as well as an interaction between Valence and Age group, *F*(1, 52) = 10.86, *p* = .002, η_p_^2^ = .17. Young and older adults were more biased towards a *yes* response for positive traits (young adults: *M* = −.36, *SE* = .08; older adults: *M* = −.53, *SE* = .08) than they were for negative traits (young adults: *M* = −.15, *SE* = .07; older adults: *M* = −.12, *SE* = .07; young adults: *p* < .001, η_p_^2^ = .33; older adults: *p* < .001, η_p_^2^ = .63). This effect of Valence on bias appears to have been larger for older adults than young adults.

### Summary of behavioural results

When indicating whether presented traits applied to themselves (in the past, present or future) or to a specified occupation, participants were faster to respond to positive than to negative traits in all conditions except past self-knowledge, regardless of age group. Both age groups were also faster to endorse positive compared with negative traits, but this effect was magnified in older adults. Young adults also endorsed more positive and less negative traits in the future self-knowledge condition compared with all other condition while the number of endorsed traits was not modulated by memory type for older adults. While both young and older adults endorsed a similar number of positive traits, older adults endorsed fewer negative traits than young adults.

In the episodic memory condition, participants were faster to correctly reject negative compared with positive traits, while there was no difference for hits. In term of accuracy during the episodic memory task, both young and older adults had more hits and less correct rejections for positive compared with negative traits. Sensitivity was higher in young than older adults while both groups showed a bias toward yes responses for positive compared with negative traits.

### Electrophysiological data

#### N400 time window (250–500 ms)

**Fig. 5:**
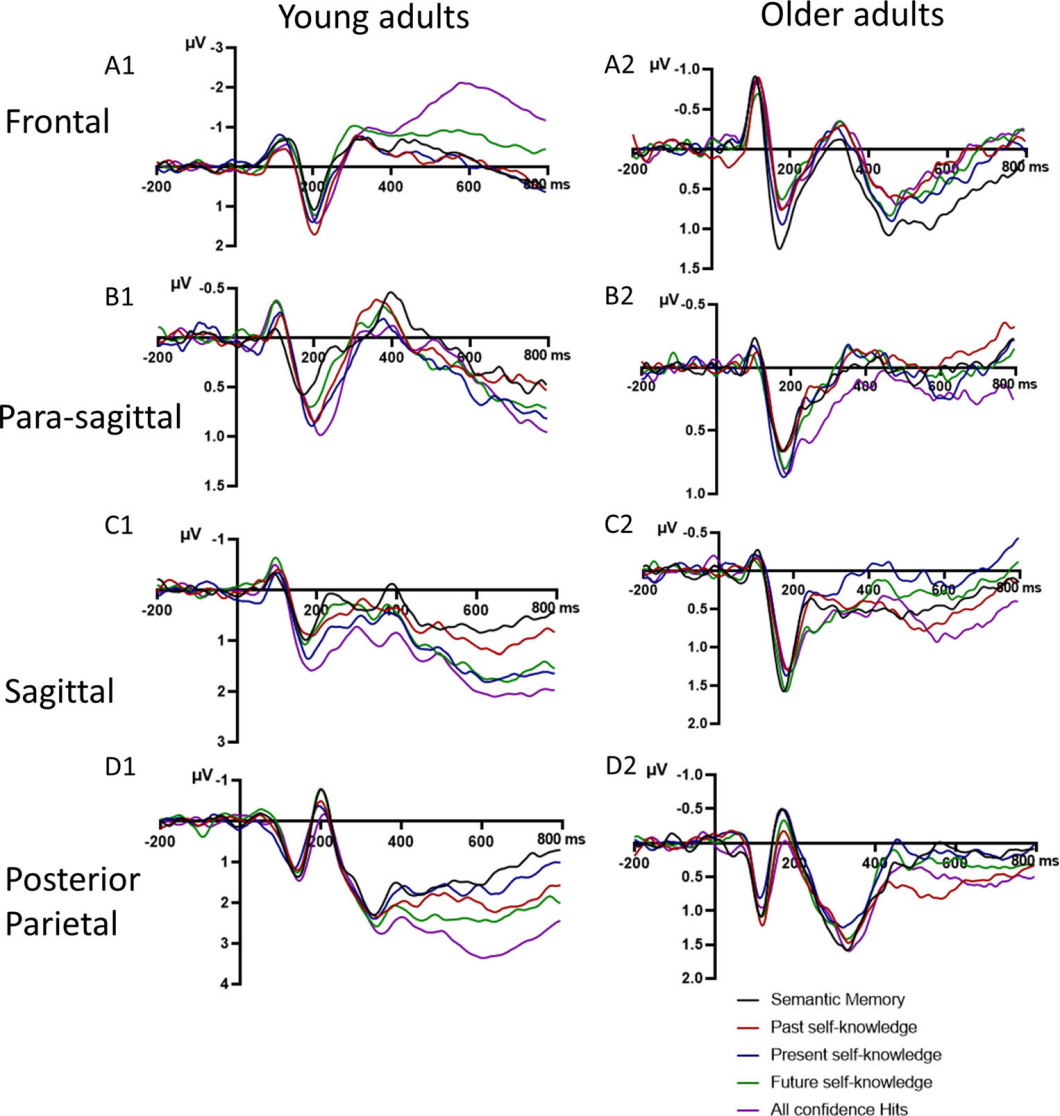
Grand average group ERPs for young (A1, B1, C1, D1; *N* = 28) and older adults (A2, B2, C2, D2; *N* = 26) of yes responses for semantic memory, personal semantics (past, present and future self-knowledge) and episodic memory (all confidence hits), over **a** frontal, **b** para-sagittal, **c** sagittal, and **d** posterior parietal sites. Negative voltage is plotted upwards. A low-pass filter of 20 Hz was applied on the grand average group data

**Fig. 6:**
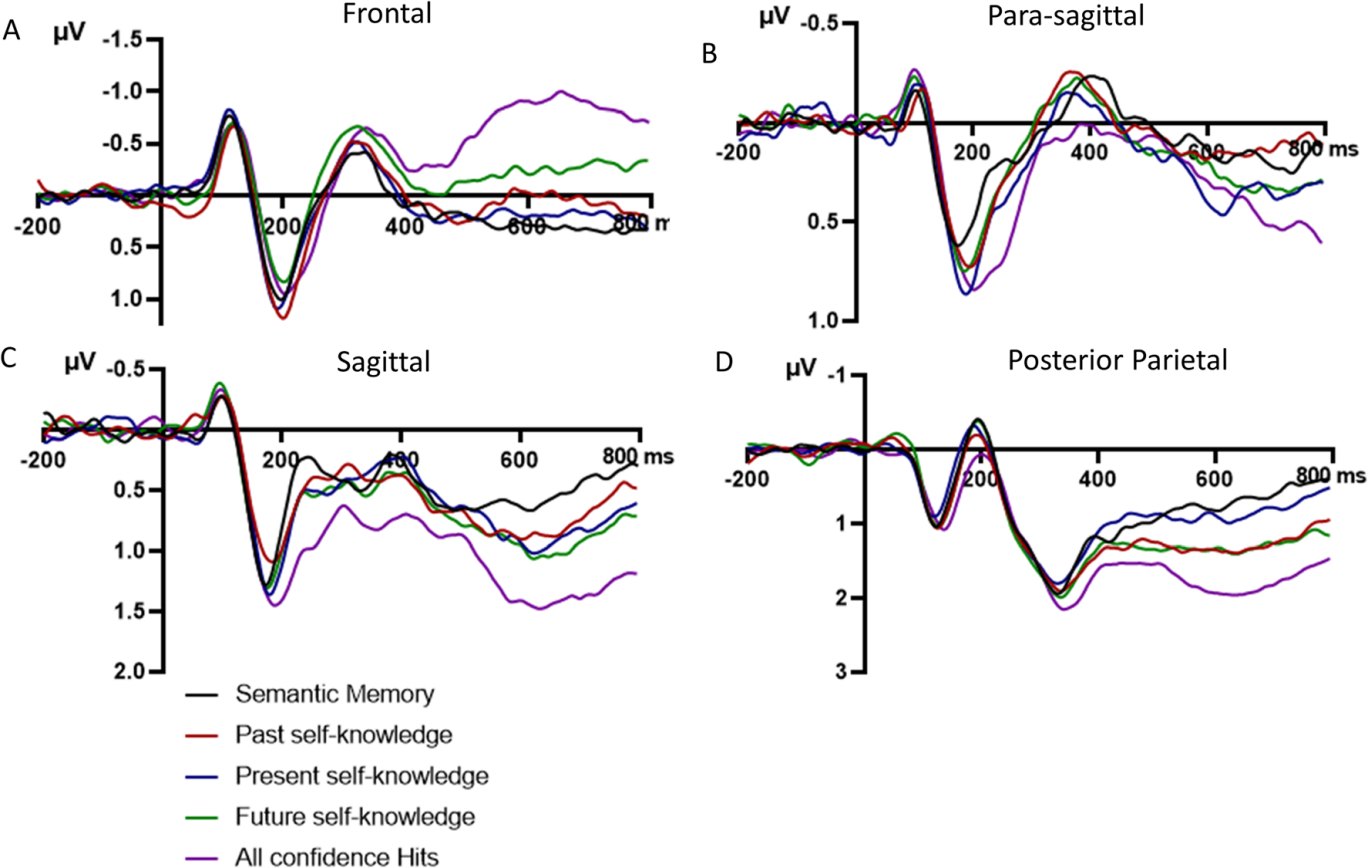
Grand average group ERPs (*N* = 54) of yes responses for semantic memory, personal semantics (past, present and future self-knowledge) and episodic memory (all confidence hits) for all participants, over **a** frontal, **b** para-sagittal, **c** sagittal, and **d** posterior parietal sites. Negative voltage is plotted upwards. A low pass filter of 20 Hz was applied on the grand average group data

**Fig. 7:**
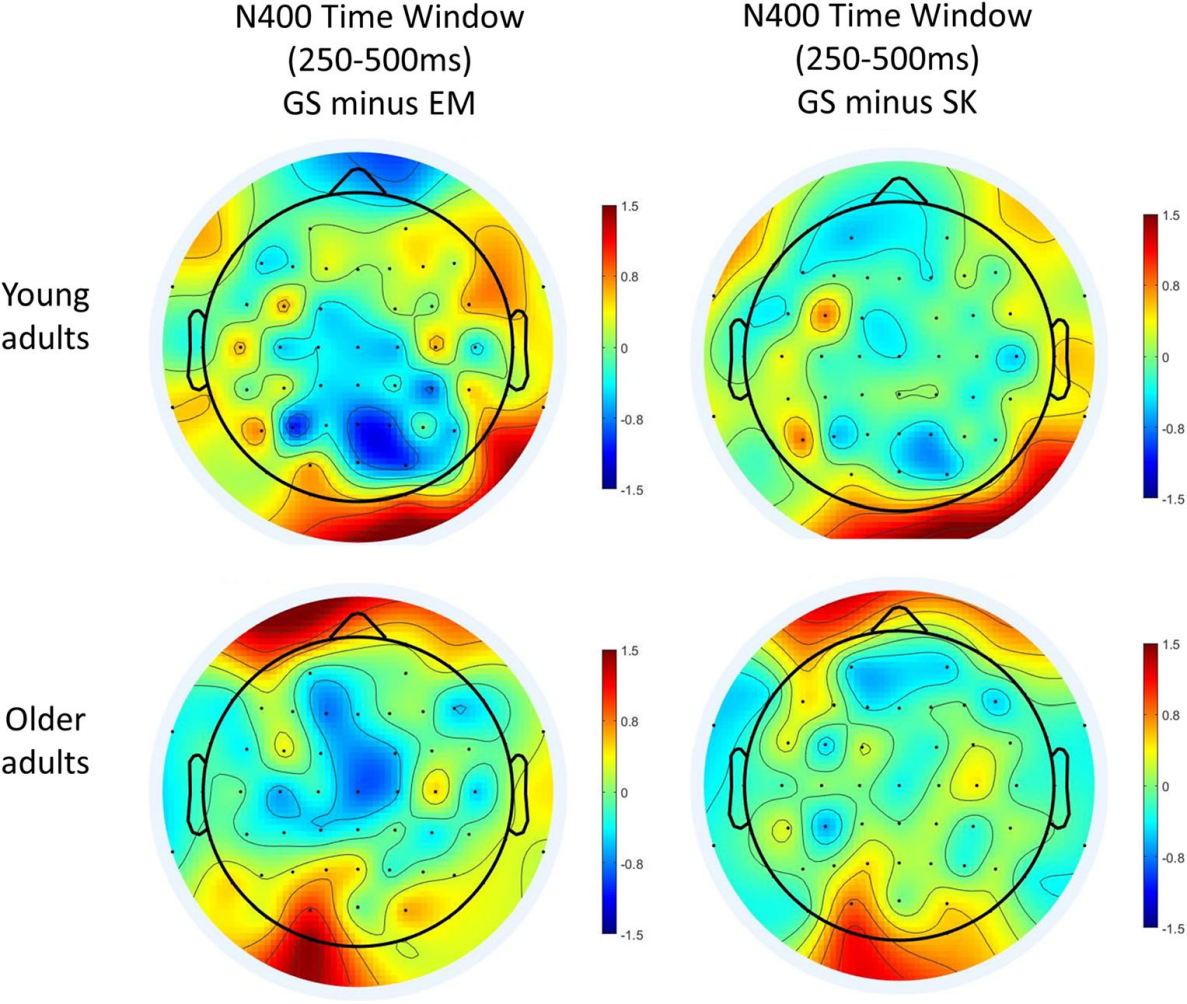
Isovoltage scalp maps for the N400 time window (250 ms to 500 ms) for *young* (top row) and older adults (bottom row). Left: Scalp maps of semantic memory (SM, yes responses) minus hits (EM). Right: Semantic memory (SM, yes responses) minus the average of all self-knowledge conditions (SK, yes responses). Scalp maps were prepared in EEGLAB (Delorme & Makeig, 2004)

We tested whether there was a difference in mean N400 amplitude over sagittal and para-sagittal sites between the memory types and whether this was modulated by age group (see Figs. 5 and 6, panels b–c, for ERP traces, and Fig. 7 for scalp maps). These mixed ANOVAs included Age group (between subject factor: young adult, older adults) and Memory type (within subject factor: general semantics, past self-knowledge, present self-knowledge, future self-knowledge, episodic memory). The para-sagittal ROI also included Hemisphere (left, right) as a within subject factor.

Over the sagittal ROI, the main effect of Memory type was significant, *F*(4, 208) = 2.46, *p* = .046, η_p_^2^ = .05, and did not interact with Age group, *F*(4, 208) = 1.18, *p* = .320, η_p_^2^ = .02. Episodic memory was less negative than general semantics (*p* = .007, Hedges’ *g* = .36), past self-knowledge (*p* = .031, Hedges’ *g* = .29), but not present self-knowledge (*p* = .216, Hedges’ *g* = .17) or future self-knowledge (*p* = .955, Hedges’ *g* = .01). Future self-knowledge was also less negative than general semantics (*p* = .033, Hedges’ *g* = .36) and past self-knowledge (*p* = .032, Hedges’ *g* = .29), and did not differ from present self-knowledge (*p* = .237, Hedges’ *g* = .17). General semantics did not differ from past (*p* = .661, Hedges’ *g* = .07) or present self-knowledge (*p* = .325, Hedges’ *g* = .15), and past and present self-knowledge did not differ from one another (*p* = .487, Hedges’ *g* = .09; see Fig. 6b–c for the ERP group data of all participants).

Over the para-sagittal subset, Memory type interacted with Hemisphere, *F*(3.48, 180.57) = 5.53, *p* = .001, η_p_^2^ = .10. Over the left hemisphere, episodic memory had a less negative amplitude compared with general semantics (*p* < .001, Hedges’ *g* = .58), past self-knowledge (*p* = .006, Hedges’ *g* = .40), present self-knowledge (*p* < .001, Hedges’ *g* = .51), and future self-knowledge (*p* = .040, Hedges’ *g* = .31). General semantics and the three self-knowledge conditions did not differ from one another (*p*s > .05, Hedges’ *g* < .22). Over the right hemisphere, future self-knowledge had a significantly less negative amplitude compared with episodic memory (*p* = .002, Hedges’ *g* = .46), past self-knowledge (*p* = .013, Hedges’ *g* = .36), and general semantics (*p* = .030, Hedges’ *g* = .32), but not present self-knowledge (*p* = .211, Hedges’ *g* = .20). Episodic memory, general semantics, and past and present self-knowledge did not differ from one another (*p*s > .05, Hedges’ *g* = .24). None of the other main effects or interactions were significant: Memory type, *F*(4, 208) = 2.07, *p* = .087, η_p_^2^ = .04; Hemisphere, *F*(1, 52) = 1.56, *p* = .217, η_p_^2^ = .03; Memory type × Age group, *F*(4, 208) = 1.90, *p* = .112, η_p_^2^ = .04; Hemisphere × Age group, *F*(1, 52) = 3.88, *p* = .054, η_p_^2^ = .07; Memory type × Hemisphere × Age group, *F*(3.47, 180.57) = 0.33, *p* = .832, η_p_^2^ = .01.

As compared with episodic memory, the scalp distribution of the general semantic condition had a classic centro-parietal distribution in the N400 time window (see Fig. 7), though the effect was also apparent over frontal sites in older adults

##### Summary of the results for the N400 time window

At sagittal sites there was a main effect of memory type with a less negative N400 amplitude for episodic memory compared with all other memory types. Future self-knowledge was also less negative compared with past self-knowledge and general semantic memory. At parasagittal sites episodic memory was less negative than all other memory types in the left hemisphere while future self-knowledge was less negative than episodic memory, past self-knowledge, and general semantic memory. There were no significant interactions between memory type and age groups for the N400.

#### LPC Time Window (500–800 ms)

We entered the mean LPC amplitude over each region of interest (ROI) in mixed ANOVAs with Age group (between subject: young adult, older adult) and Memory type (within subject: general semantics, past self-knowledge, present self-knowledge, future self-knowledge, episodic memory). The posterior parietal, para-sagittal, and frontal ROIs also included a Hemisphere factor (within subject: left, right).

##### Posterior parietal

**Fig. 8:**
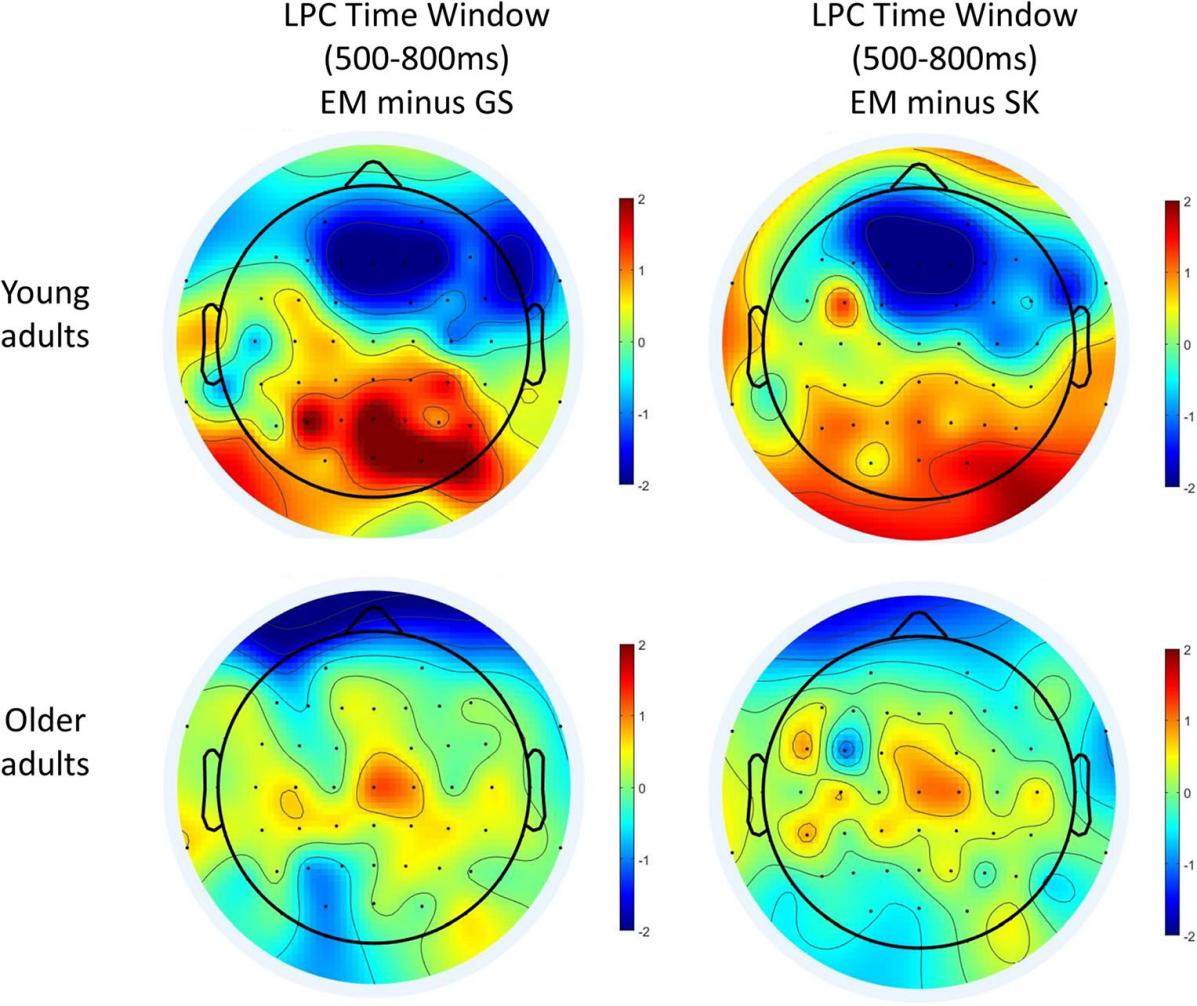
Isovoltage scalp maps for the LPC time window (500 ms to 800 ms) in young (top row) and older adults (bottom row). Left: Scalp maps of hits (EM) minus semantic memory (SM, yes responses). Right: hits (EM) minus the average of all self-knowledge conditions (SK, yes responses). Scalp maps were prepared in EEGLAB (Delorme & Makeig, 2004)

We tested whether there was a difference in mean LPC amplitude over posterior parietal sites (see Figs. 5 and 6, panel D for ERP traces, and Fig. 8 for scalp maps). The main effect of Memory type, *F*(4, 208) = 4.31, *p* = .002, η_p_^2^ = .08, and the interaction between Memory type and Age group, *F*(4, 208) = 6.19, *p* < .001, η_p_^2^ = .11, were significant while the main effect of Hemisphere, *F*(1, 52) = 1.65, *p* = .205, η_p_^2^ = .03, and the interaction between Hemisphere and Age group, *F*(1, 52) = 0.30, *p* = .588, η_p_^2^ = .01, were not. Age group, Memory type, and Hemisphere also interacted, *F*(3.12, 162.11) = 3.67, *p* = .013, η_p_^2^ = .07. To investigate this interaction further, we ran repeated measures ANOVAs for each age group separately with Memory and Hemisphere as factors.

For the older adults, there was no main effect of Memory, *F*(2.95, 73.71) = 0.68, *p* = .567, η_p_^2^ = .03, no main effect Hemisphere, *F*(1, 25) = 0.43, *p* = .521, η_p_^2^ = .02, and no interaction between Memory and Hemisphere, *F*(2.38, 59.51) = 1.08, *p* = .355, η_p_^2^ = .04 (see Fig. 4).

For young adults, the main effect of Memory was significant, *F*(4, 108) = 7.80, *p* < .001, η_p_^2^ = .22; see Fig. 5, and the effect of Memory depended on Hemisphere, *F*(4, 108) = 3.28, *p* = .014, η_p_^2^ = .11.

General semantics was less positive than all other memory conditions (past self-knowledge: *p* = .009, Hedges’ *g* = .44; present self-knowledge: *p =* .028, Hedges’ *g* = .28; future self-knowledge*: p =* .004, Hedges’ *g* = .45; episodic memory: *p <* .001, Hedges’ *g* = .70) over the left hemisphere, whereas it was only less positive than episodic memory over the right hemisphere (*p* = .003, Hedges’ *g* = .51; past self-knowledge: *p* = .958, Hedges’ *g* = .01; present self-knowledge: *p* = .468, Hedges’ *g* = .10; future self-knowledge: *p* = .200, Hedges’ *g* = .22). The three self-knowledge conditions were less positive than episodic memory over both hemispheres (left—past: *p* = .013, Hedges’ *g* = .31, present: *p* = .014, Hedges’ *g* = .36, future: *p* = .012, Hedges’ *g* = .26; right—past: *p <* .001, Hedges’ *g* = .56, present: *p* < .001, Hedges’ *g* = .65, future: *p* = .033, Hedges’ *g* = .27). Past self-knowledge did not differ from present or future self-knowledge over both hemispheres (left—present: *p* = .446, Hedges’ *g* = .09, future: *p* = .766, Hedges *g* = .04; right—present: *p* = .372, Hedges’ *g* = .12, future: *p* = .113, Hedges’ *g* = .24). Present self-knowledge was significantly less positive than future self-knowledge over the right hemisphere (*p* = .034, Hedges’ *g* = .34), but not the left hemisphere (*p* = .311, Hedges’ *g* = .12). The main effect of Hemisphere was not significant, *F*(1, 27) = 1.29, *p* = .267, η_p_^2^ = .05.

Even though, the LPC or parietal old-new effect is typically investigated at posterior parietal sites, differences in scalp distributions are sometimes observed in ageing (e.g., J. H. Ford & Kensinger, 2019; Horne et al., 2020; Newsome et al., 2012). We thus tested whether older adults could have similar effects as young adults in the LPC time window at other scalp sites (frontal, sagittal, para-sagittal):

##### Frontal

At this ROI, the main effect of Memory was significant, *F*(4, 208) = 9.81, *p* < .001, η_p_^2^ = .16, and depended on the Age group, *F*(4, 208) = 8.18, *p* < .001, η_p_^2^ = .14. The main effect of Hemisphere was also significant, *F*(1, 52) = 4.27, *p* = .044, η_p_^2^ = .08.

None of the Memory types differed from one another for older adults (*p*s > .05; see Fig. 4a), whereas some differences emerged for young adults (see Fig. 5a). In young adults, episodic memory was less positive than all other Memory types (*p*s < .001, Hedges’ *g* > .62). Future self-knowledge was less positive than present self-knowledge (*p* = .038, Hedges’ *g* = .25) and past self-knowledge (*p* = .026, Hedges’ *g* = .30), but not general semantics (*p* = .119, Hedges’ *g* = .22). Past and present self-knowledge did not differ from one another (*p* = .969, Hedges’ *g* < .01), nor from general semantics (past: *p* = .572, Hedges’ *g* = .07; present: *p* = .671, Hedges *g* = .05). Further, the main effect of Hemisphere was significant, *F*(1, 52) = 4.27, *p* = .044, η_p_^2^ = .08; the mean LPC amplitude was less positive for the left (*M* = −0.27, *SE* = .17) than right hemisphere (*M* = −0.02, *SE* = .18). None of the other effects were significant: Memory type × Age group × Hemisphere, *F*(4, 208) = 0.91, *p* = .457, η_p_^2^ = .02; Memory × Hemisphere, *F*(4, 208) = 1.48, *p* = .211, η_p_^2^ = .03; Age group × Hemisphere, *F*(1, 52) = 3.59, *p* = .064, η_p_^2^ = .07.

##### Para-sagittal

None of the interactions with Age group were significant for the para-sagittal ROI: Memory type and Age group, *F*(4, 208) = 0.93, *p* = .447, η_p_^2^ = .02; Hemisphere and Age group *F*(1, 52) = 1.22, *p* = .275, η_p_^2^ = .02; and Memory, Hemisphere, and Age group, *F*(4, 208) = 0.53, *p* = .714, η_p_^2^ = .01. The main effects of Memory type, *F*(4, 208) = 1.56, *p* = .186, η_p_^2^ = .03, and of Hemisphere, *F*(1, 52) = 3.16, *p* = .081, η_p_^2^ = .06, were not significant but the interaction between Memory type and Hemisphere was, *F*(4, 208) = 4.75, *p* = .001, η_p_^2^ = .08. None of the Memory conditions differed from one another over the right hemisphere, *p*s > .05 (Hedges’ *g* < .22). Over the left hemisphere, general semantic and all self-knowledge conditions were significantly less positive than episodic recognition (general semantics: *p* < .001, Hedges’ *g* = .56; past self-knowledge: *p* = .005, Hedges’ *g* = .42; present self-knowledge: *p* = .010, Hedges’ *g* = .41; future self-knowledge: *p* = .004, Hedges’ *g* = .45). General semantics, past self-knowledge, present self-knowledge, and future self-knowledge did not differ from one another (*p*s > .05, Hedges’ *g* < .19).

##### Sagittal

At this ROI, the effect of Memory was also significant, *F*(4, 208) = 6.56, *p <* .001, η_p_^2^ = .11, and depended on Age group, *F*(4, 208) = 2.53, *p* = .042, η_p_^2^ = .05. For older adults, only present self-knowledge was less positive than episodic memory (*p* = .006, Hedges’ *g* = .32). None of the other comparisons were significant (*p* > .05, Hedges’ *g* < .29, see Fig. 4c). For young adults (see Fig. 5c), there were several additional differences. General semantics was less positive than present self-knowledge (*p* = .003, Hedges’ *g* = .44), future self-knowledge (*p* < .001, Hedges’ *g* = .53) and episodic memory (*p* < .001, Hedges’ *g* = .57), but not past self-knowledge (*p* = .260, Hedges’ *g* = .15). Past self-knowledge was also less positive than present self-knowledge (*p* = .022, Hedges’ *g* = .28), future self-knowledge (*p* = .003, Hedges’ *g* = .37), and episodic memory (*p* = .003, Hedges’ *g* = .42). Present self-knowledge did not differ from either future self-knowledge (*p* = .518, Hedges’ *g* = .08) or episodic memory (*p* = .192, Hedges’ *g* = .15), which in turn were also not significantly different from one another (*p* = .581, Hedges’ *g* = .07).

As compared with semantic memory, the scalp distribution of the episodic memory condition had a classic posterior-parietal distribution in the LPC time window in young adults, with local maxima at electrodes Pz and P3 but also over right temporo-occipital sites (see Fig. 8). In contrast, in older adults, the effect was reduced in magnitude and mainly apparent at central sites.

##### Summary of the results for the LPC time window

Across both age groups, episodic memory was associated with a more positive LPC amplitude compared with all other conditions in the left hemisphere at parasagittal sites. For older adults, the LPC amplitude in the present self-knowledge condition was less positive than the LPC amplitude in the episodic memory condition at sagittal sites, while there were no significant differences between memory conditions at posterior parietal or frontal sites. For young adults, the general semantic condition was associated with a reduced LPC amplitude compared with all other memory conditions in the left and episodic memory in the right hemisphere at posterior parietal sites. At frontal sites, episodic memory was associated with a less positive LPC amplitude compared with all other conditions. Additionally, future self-knowledge was also associated with a reduced LPC amplitude compared with the past and present self-knowledge conditions. At sagittal sites, the general semantic memory and present self-knowledge conditions were associated with a less positive LPC amplitude compared with all other conditions but did not differ from each other.

### Manipulation check

#### LPC (500–800 ms)

**Fig. 9:**
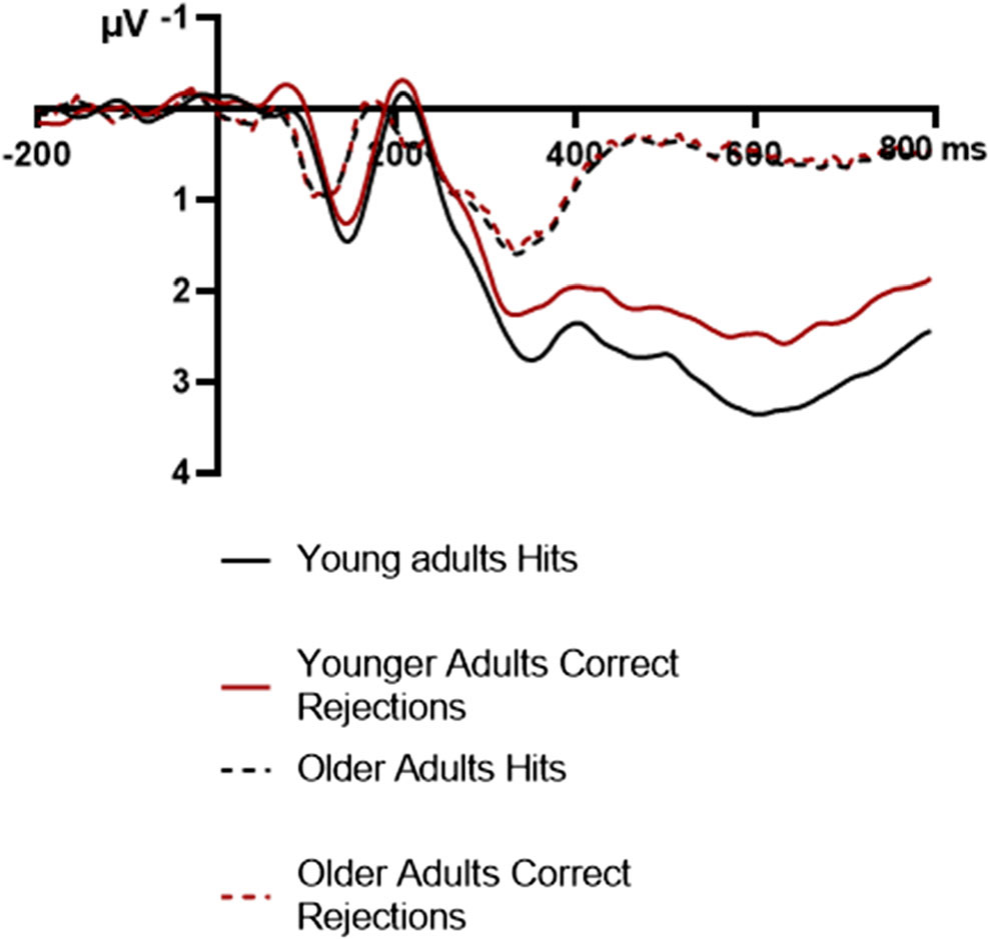
Grand-averaged ERPs of hits and correct rejections for older (*N* = 26) and young (*N* = 28) adults separately at posterior parietal sites. Negative voltage is plotted upward. Grand-averages were low-pass filtered at 20 Hz

**Fig. 10:**
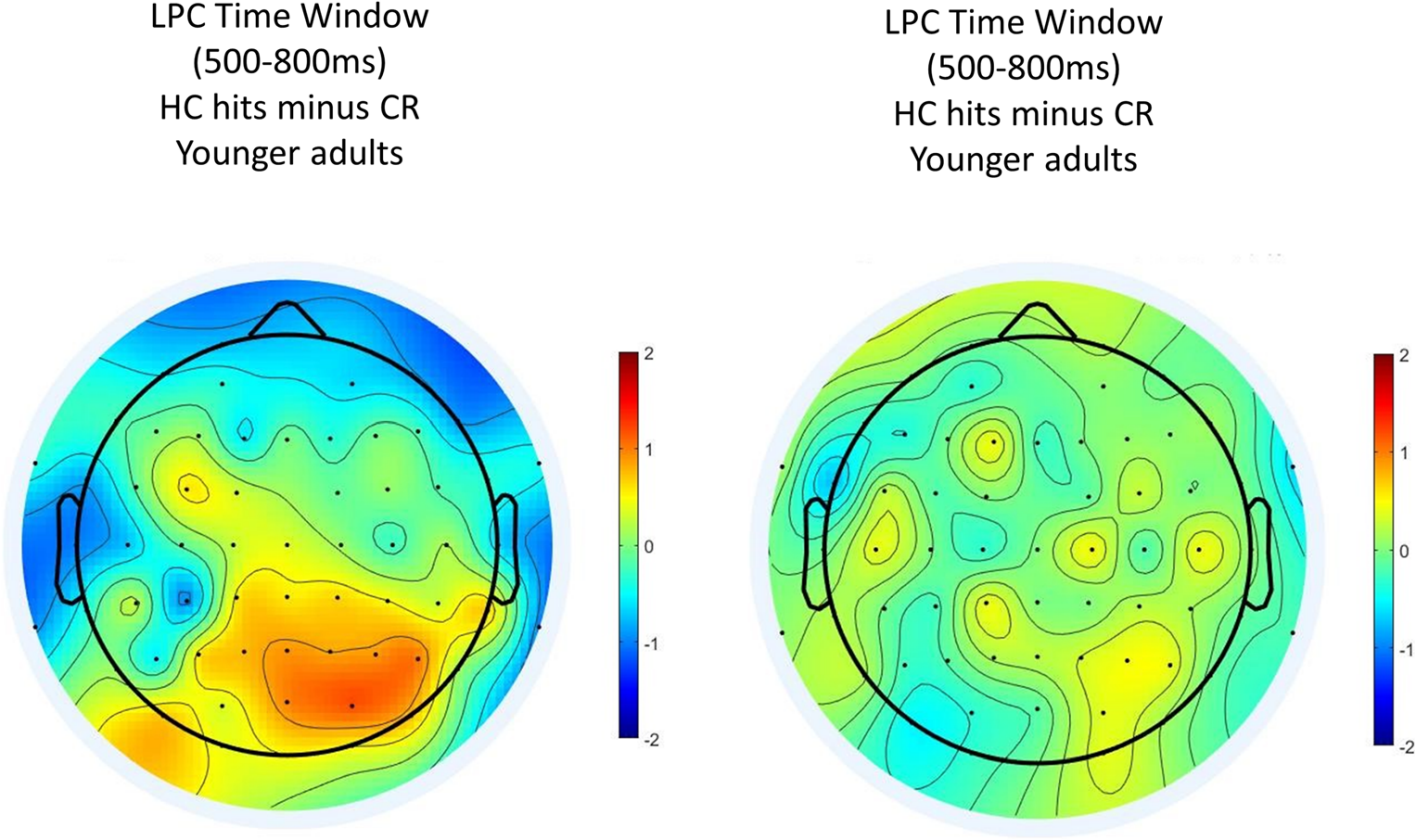
Isovoltage scalp maps in the LPC time window (500 ms to 800 ms) for the old–new effect (hits minus correct rejections) for young adults at the left, and older adults at the right. Scalp maps were prepared in EEGLAB (Delorme & Makeig, 2004)

A well-established finding is the more positive LPC amplitude for hits compared with correct rejections, also known as the parietal old/new effect (Rugg & Curran, 2007). We ran a 2 × 2 × 2 mixed ANOVA with Response type (hits, correct rejections), and Age group, and Hemisphere as above (see Fig. 9 for ERP traces and Fig. 10 for scalp maps). The main effect of Response type, *F*(1, 52) = 13.40, *p* < .001, η_p_^2^ = .21, and the interaction between Response type and Age group were significant, *F*(1, 52) = 11.15, *p =* .002, η_p_^2^ = 18. For older adults, there was no significant difference between hits and correct rejections (*p* = .824, η_p_^2^ < .01) while for young adults the LPC was increased for hits compared with correct rejections (*p* < .001, η_p_^2^ = .33). None of the other effects were significant: Hemisphere, *F*(1, 52) = 1.25, *p* = .269, η_p_^2^ = .02; Hemisphere × Age group, *F*(1, 52) = 0.71, *p* = .404, η_p_^2^ = 01; Response type × Hemisphere, *F*(1, 52) = 2.28, *p* = .137, η_p_^2^ = .04; Response type × Hemisphere × Age group, *F*(1, 52) = 0.20, *p* = .657, η_p_^2^ < .01. In summary, while there was an increased LPC amplitude for hits compared with correct rejections in young adults, there was no difference in older adults.

#### Correlations between Neuropsychological and Electrophysiological measures

Older adults with good episodic memory function might be able to recruit episodic processing to think about a past and future self. We hypothesized that the recruitment of episodic processes could produce a representation of distant selves that is more precise and contextually specific than the present self. This rich representation of the future self, well anchored in a distant time, might differentiate distant selves from the present self, behaviourally and neurally. If so, those with better episodic memory function might (1) show a greater mean difference in percentage of positive or negative traits between distant and present times (mean of future minus present and past minus present); (2) show a greater mean difference in LPC amplitude between distant and present self-knowledge (mean of future minus present and past minus present). Executive functions are also sensitive to ageing and may explain why some older adults would have reduced cognitive resources to maintain the goal of the task in mind—including temporal orientation—while performing a relatively fast-paced task. Although deficits in executive performance might explain some of the variance in performance or neural activity, our core hypotheses concerned episodic memory function and we included executive functions mostly as comparison. We also tested whether episodic memory function related with an increased parietal old/new effect. We had 5 key correlations with a directional hypothesis.

**Fig. 11:**
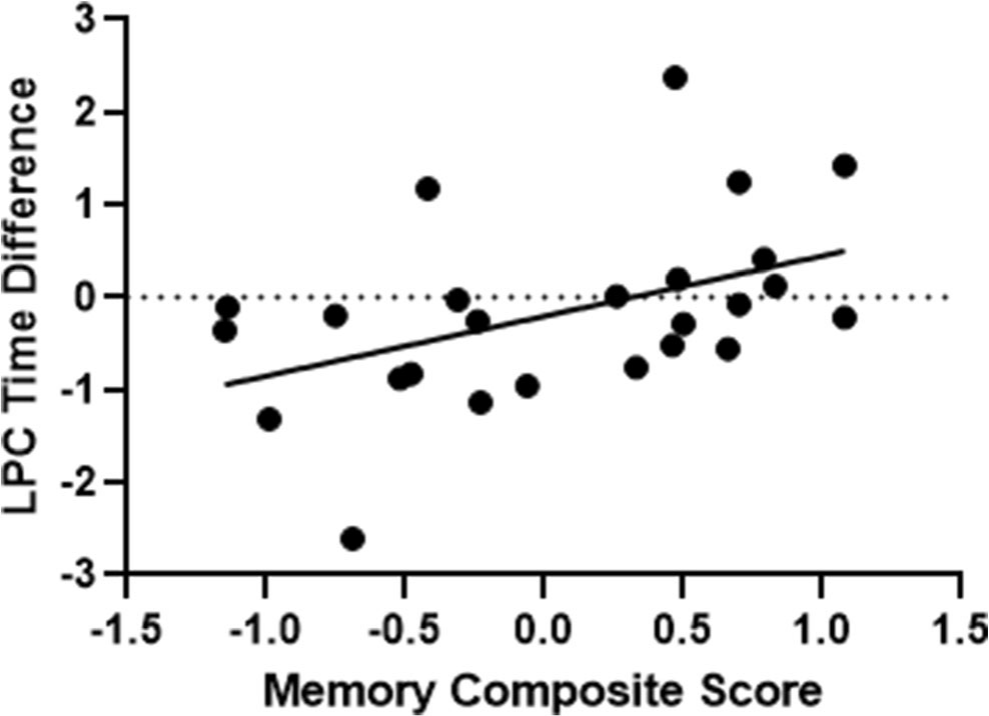
Scatter plot of the correlation between the memory composite score (based on logical memory and Rey–Osterrieth complex figure delayed recall performance) and the LPC time difference (mean of future minus present and past minus present) in older adults, including the trend line (solid black line)

Although no correlation was significant (see Table 1) after correction for multiple comparison (Holm–Bonferroni), one *r* value represented a medium-to-large effect size and had a *p* value of .012 (slightly above the cut-off of .01; see Table 1). Participants with a better episodic memory function had a larger positive LPC difference associated with the temporal orientation of self-knowledge (i.e., larger and more positive LPC amplitude for distant times than now, as seen for young adults on average). We conducted additional partial correlations to possibly minimize the effect of confounding variables: age, symptoms of depression, cognitive status (MoCA), and the processing speed composite. Controlling for age increased the strength of the correlation between the episodic memory composite and mean LPC time difference to, *r*(22) = .71, and lowered the *p* value to .000044. The relation persisted in the additional partial correlations, which hovered around the values of the full correlation, symptoms of depression: *r*(22) = .45, *p* = .014; cognitive status: *r*(22) = .46, *p* = .012; processing speed: *r*(22) = .45, *p* = .013. In summary, participants with a better episodic memory function had a larger positive LPC difference associated with the temporal orientation of self-knowledge and controlling for age increased the strength of this relation (see Fig. 11).

**Table 1:** Correlation coefficients between neuropsychological tests, LPC amplitude, and behaviour in older adults

	1	2	3	4	5
1-Episodic memory composite	–				
2-Executive function composite	*r* = −.03 *p* = .446 (*n* = 25)	–			
3-Behaviour mean time difference	*r* = −.16 *p* = .221 (*n* = 25)	*r* = .04 *p* = .432 (*n* = 25)	–		
4- LPC mean time difference	*r* = .45* *p* = .012 (*n* = 25)	*r* = −.07 *p* = .363 (*n* = 25)	*r* = −.12 *p* = .285 (*n* = 26)	–	
5-LPC difference between hits and CR	*r* = −.15 *p* = .235 (*n* = 25)	*r* = −.14 *p* = .245 (*n* = 25)	*r* = .25 *p* = .105 (*n* = 26)	*r* = −.15 *p* = .231 (*n* = 26)	–

*Note*. *p < .05 (one-tailed)

## Discussion

Recent studies have revealed that the neural correlates of self-knowledge could be dissociated from those of general semantic and episodic memory in young adults (Tanguay et al., 2018; Tanguay et al., 2020). However, studies of “dedifferentiation” or loss of distinctiveness of neural representations in older adults (Cabeza et al., 2018; Koen et al., 2020; Koen & Rugg, 2019), including in semantic and episodic memory tasks (Park et al., 2004; St-Laurent et al., 2011; St-Laurent et al., 2014; Zheng et al., 2018), suggest that the neural correlates of self-knowledge might be less distinct from those of semantic and episodic memory in older adults. The results of the present ERP study are consistent with this hypothesis. The amplitude of the LPC component, associated with episodic recollection, differentiated the self-knowledge, general semantic and episodic conditions in young adults, but not in older adults. However, in older adults, participants with higher composite episodic memory scores (based on logical memory and Rey–Osterrieth complex figure delayed recall performance) had more differentiated LPC amplitudes across the self-knowledge experimental conditions. Our findings thus suggest that declarative memory subtypes are less distinct in ageing, but that that the amount of differentiation varies with episodic memory function.

During the encoding tasks, both young and older adults were faster to endorse a trait if it was positive, but slower if it was negative. When considering the percentage of “yes” responses, we observed that young adults endorsed more positive traits and less negative traits in the future compared with the present and past self-knowledge conditions, and also compared with other people’s traits (see also Tanguay et al., 2018), consistent with an optimism bias (Kanten & Teigen, 2008; Sharot, 2011). In contrast, in older adults, the temporal orientation of self-knowledge, or whether knowledge concerned the self or other people, did not influence the percentage of endorsed positive and negative traits. Older adults had thus a less differentiated processing of the traits across the various experimental conditions. Note however, that consistent with a good self-esteem (Orth et al., 2018), and a general high level of optimism (Borges & Dutton, 1976) or a “positivity effect” in ageing (Tomaszczyk & Fernandes, 2012, 2013), the ratio of “yes” responses for positive traits was high across categories in older adults (close to 70%) and similar to the highest ratio of “yes” responses observed for young adults in the future condition. Conversely, older adults were significantly less likely to endorse negative traits across all memory types compared with young adults. Also compatible with this positivity effect in ageing, in the recognition task, older adults had a higher hit rate for positive than negative traits, and their hit rate for positive traits (83%) was similar to the average hit rate observed in young adults (84%; for whom the valence effect in recognition performance was weaker).

For young adults, ERP analyses revealed that, at posterior parietal sites where it is typically measured (Rugg & Curran, 2007; Wilding & Ranganath, 2012), the amplitude of the LPC was maximal for the episodic condition, intermediate for the self-knowledge conditions and minimal for the general semantic condition (see also Tanguay et al., 2018, 2020). Over right hemisphere sites, future self-knowledge also produced larger LPC amplitudes than present self-knowledge, consistent with the idea that time perspective modulates how self-knowledge is accessed in young adults, and that thinking about future traits may have involved increased episodic processing (Tanguay et al., 2018; Tanguay et al., 2020). None of these differences were significant in the older adults. Thus, consistent with our hypotheses, there was no difference across memory types, and temporal orientation did not modulate the mean LPC amplitudes in older adults. The averaged difference in LPC amplitude between the temporally distant (i.e., past and future) and present self-knowledge conditions were related with episodic memory function in older adults. Those older adults with better episodic memory function had larger LPC amplitude differences between the temporally distant and present self-knowledge conditions. The neural correlates of these memory types might be more differentiated in these older adults, and they might better be able to recruit episodic processes when thinking about distant selves. Additionally, at posterior parietal sites, the old-new effect (difference between hits and correct rejections) was only significant in young adults. We would expect similar findings if we replaced the lab-based episodic memory task with an autobiographical episodic memory task (see, for example, Johnson et al., 2011; Renoult et al., 2016), but this should be tested. Even though the small effect size (η_p_^2^ = .03) for the mean LPC amplitude over the posterior ROI signifies that finding such an effect of Memory in older adults would be challenging, if possible at all, the results of our study should be replicated because of the novelty and exploratory nature of some analyses. More generally, the idea of a recruitment of episodic processes for semantic forms of memory in ageing and in conditions with severe decline in episodic memory is little studied (but see Duff et al., 2019; Grilli & Verfaellie, 2016). The factors influencing the engagement of episodic processes (e.g., spatiotemporal context) and their role should be further examined.

As different scalp distributions are sometimes observed in older adults, as compared with young adults (e.g., Ford & Kensinger, 2019; Horne et al., 2020; Newsome et al., 2012), we also tested whether older adults may have similar effects as young adults in the LPC time window at other scalp sites (frontal, sagittal, para-sagittal). At frontal sites, there were some significant modulation of voltage amplitudes in the LPC time window by memory type (episodic memory differing from all other memory types, and future self-knowledge from present and past self-knowledge) but again only in young adults. At sagittal sites, voltage amplitudes in the LPC time window were more positive for episodic memory than for present self-knowledge in older adults. In young adults, additional differences between memory types were observed, such as differentiation between episodic memory and general semantics, as well as present and future self-knowledge. In contrast, at left para-sagittal sites, some differences between memory types, albeit more limited than at posterior parietal sites, were found in both age groups. At these electrode sites, voltage amplitudes in the LPC time window were more positive for the episodic condition than for all other conditions.

The fact that a reduced differentiation of memory types was observed at sagittal and left para-sagittal sites in both age groups (essentially differences in LPC amplitudes between episodic memory and all other conditions) argue against the idea that these effects would be a form of compensation in older adults (Cabeza et al., 2018). Compensation by selection or reorganization occurs when older adults recruit processes or brain regions that young adults do not (Cabeza et al., 2018). Similarly, some limited N400 differences between memory types were also observed in both age groups . This is consistent with a general preservation of semantic memory in ageing and with relatively similar N400 *effects* (the difference between two experimental conditions) in young and older adults (Wlotko et al., 2010). However, even if N400 effects are significant in older adults (and thus conditions are significantly differentiated), the magnitude of the difference between conditions is sometimes reduced (Federmeier & Kutas, 2005; Federmeier et al., 2003; Ford et al., 1996). Here, for both young and older adults, N400 amplitudes were maximal for general semantics and self-knowledge and clearly smaller for episodic memory. At certain electrode sites (sagittal and right para-sagittal), future self-knowledge also produced less negative amplitudes than general semantics. Even though the magnitude of the N400 difference between memory types appeared slightly reduced in older as compared with young adults (see scalp maps of Fig. 7), there were no significant interaction between memory types and age. Taken together, these results are thus consistent with the hypothesis that a dedifferentiation of subtypes of declarative memory with age is more apparent for the LPC than for the N400. It is possible that in the present task the N400 findings reflect “maintenance” (Nyberg et al., 2012) of semantic processes in older adults that are sufficient to perform the task. More generally, our findings are consistent with a semanticization of declarative memory in older adults (Levine et al., 2002; Renoult et al., 2020; St Jacques & Levine, 2007) and a decline of episodic memory function in ageing (Alghamdi & Rugg, 2020; Cansino, 2009; Tromp et al., 2015). Hence, self-knowledge, like events, might be represented in more generic terms and might be more abstracted from contexts in ageing. Trait knowledge may be slower to update and may become inaccurate (as research on patients with episodic memory deficits suggests; reviewed in Strikwerda-Brown et al., 2019).

Interestingly, there were no significant difference in N400 amplitude between general semantics and past or present self-knowledge in the present study (present self-knowledge arguably constitutes the most straightforward comparison to general knowledge, as the general semantics condition did not include any mention of time), similar to previous findings by Tanguay et al. (2020). Similarly, in a study by Coronel and Federmeier (2016), N400 amplitudes were found to be very similar for self-knowledge and general knowledge, even if there was a statistical trend for personal knowledge to produce smaller amplitudes. In the study of Tanguay et al. (2018), general semantics produced larger N400 amplitudes than all self-knowledge conditions over sagittal sites, but not over para-sagittal electrode sites (and no effect over sagittal sites in Tanguay et al., 2020). Future studies are thus required to clarify how N400 may differentiate personal and general knowledge and whether the small differences that have been reported in some previous studies are due to imperfect matching between conditions or to some genuine differences in how personal and general knowledge is processed. This latter possibility would be consistent with the finding that other types of personal semantics, like knowledge of autobiographical facts and memories of repeated events, were reported to differ in N400 amplitude from knowledge of general facts (Renoult et al., 2016).

One potential limitation of the present experimental design is that we did not have enough trials per condition to differentiate ERP effects according to response and emotional valence. However, as reported in our previous study, the effect of temporal orientation on LPC amplitude did not depend on the valence of the traits (Tanguay et al., 2020). Another potential limitation of the present design, and of studies using similar paradigms, is that the effects of time perspective may not be perfectly equivalent in young and older adults, as older adults typically perceive that their traits change less through time and traits indeed become more stable with ageing (Rutt & Lockenhoff, 2016). Further, older adults may have experienced and foresee experiencing fewer major life events than young adults with a 5-year period; these turning points may particularly be important to inform knowledge about a distant self. A possible alternative, at least for the past condition, may be to ask participants of each age group to think about a specific age (e.g., 15) or a certain life transition. However, using such experimental designs, the remoteness of the time period would no longer be matched across age groups, and remoteness is known to affect LPC effects (Roberts et al., 2013; Tsivilis et al., 2015). Moreover, it is important to note that even though a different processing of temporal perspective in ageing could have affected corresponding LPC effects, it would not have applied to the LPC old-new effect, which was also reduced in older adults. Crucially, even though, a clear differentiation of memory types was only observed in young adults at posterior parietal sites, older adults participants with higher composite episodic memory scores had a greater LPC effect of time perspective. Taken together, these findings are consistent with the fact that age-related neural dedifferentiation may be material and region specific (Koen et al., 2020; Koen & Rugg, 2019), and suggest that declarative memory subtypes are less distinct in ageing, but that that the amount of differentiation varies with episodic memory functions.
